# Carboxypeptidase A4 negatively regulates HGS-ETR1/2-induced pyroptosis by forming a positive feedback loop with the AKT signalling pathway

**DOI:** 10.1038/s41419-023-06327-5

**Published:** 2023-12-04

**Authors:** Luoling Wang, Rilin Deng, Shuishun Chen, Renyun Tian, Mengmeng Guo, Zihao Chen, Yingdan Zhang, Huiyi Li, Qian Liu, Songqing Tang, Haizhen Zhu

**Affiliations:** 1 Institute of Pathogen Biology and Immunology, College of Biology, State Key Laboratory of Chemo/Biosensing and Chemometrics, Hunan University, Changsha, 410082 China; 2 Key Laboratory of Tropical Translational Medicine of Ministry of Education, Department of Pathogen Biology, Institute of Pathogen Biology and Immunology, School of Basic Medicine and Life Science, The University of Hong Kong Joint Laboratory of Tropical Infectious Diseases, Hainan Medical University, Haikou, 571199 China

**Keywords:** Cell death and immune response, Cancer microenvironment

## Abstract

Pyroptosis, a mode of inflammatory cell death, has recently gained significant attention. However, the underlying mechanism remains poorly understood. HGS-ETR1/2 is a humanized monoclonal antibody that can bind to DR4/5 on the cell membrane and induce cell apoptosis by activating the death receptor signalling pathway. In this study, by using morphological observation, fluorescence double staining, LDH release and immunoblot detection, we confirmed for the first time that HGS-ETR1/2 can induce GSDME-mediated pyroptosis in hepatocellular carcinoma cells. Our study found that both inhibition of the AKT signalling pathway and silencing of CPA4 promote pyroptosis, while the overexpression of CPA4 inhibits it. Furthermore, we identified a positive regulatory feedback loop is formed between CPA4 and AKT phosphorylation. Specifically, CPA4 modulates AKT phosphorylation by regulating the expression of the AKT phosphatase PP2A, while inhibition of the AKT signalling pathway leads to a decreased transcription and translation levels of CPA4. Our study reveals a novel mechanism of pyroptosis induced by HGS-ETR1/2, which may provide a crucial foundation for future investigations into cancer immunotherapy.

## Introduction

Tumour necrosis factor-associated apoptosis-inducing ligand (TRAIL/Apo2L) is a type II transmembrane protein belonging to the tumor necrosis factor superfamily [1]. This molecule exhibits the ability to selectively induce apoptosis in a diverse range of tumour cells but not in normal cells. Consequently, TRAIL holds promise as a potential protein-based therapeutic drug for the treatment of tumours. To date, five distinct TRAIL receptors have been identified on the cell surface, which can be categorized into three groups: death receptor (TRAIL1/2, also known as DR4/5), decoy receptor (TRAIL3/4, also known as DcR1/2), and osteoprotegerin (OPG) [2]. Previous studies have shown that the binding of TRAIL to death receptors, the only one of the three receptor types that transmits death signals, induces apoptosis of tumour cells [3]. The extracellular region of the decoy receptor possesses a cysteine-rich repeat sequence that exhibits significant homology to the death receptor. However, due to the absence of the death domain, the decoy receptor cannot transmit the death signal despite its ability to bind to TRAIL. OPG is an osteoclast suppressor whose main function is to reduce the number of osteoclasts, increase bone density and promote bone deposition [4].

The molecular mechanism underlying TRAIL-induced cell apoptosis has been well studied. When TRAIL binds to the death receptor DR4/5, it induces the binding of the death domain (DD) of the cytoplasmic segment of the death receptor with the C-terminal DD of the Fas-associated protein with death domain (FADD) [5]. FADD binds to procaspase-8 via its N-terminal death effector domain (DED) to form the DR4/5/FADD/procaspase-8 death-inducing signalling complex (DISC), which promotes the self-catalysis of procaspase-8 to activate caspase-8 [6, 7]. With the activation of caspase-8, the transmission of apoptosis signals can be initiated, and the downstream caspase signalling cascade can be activated, ultimately inducing apoptosis [8].

HGS-ETR1 (mapatumumab) and HGS-ETR2 (lexatumumab) are humanized monoclonal antibodies developed by Human Genome Sciences (HGS) that specifically bind to DR4/5 protein [9–11]. HGS-ETR1/2 are agonist antibodies that mimic the activity of natural TRAIL and they have been demonstrated to possess potent antitumour activity by activating the death signalling pathway and inducing cell death through binding with DR4/5 [12, 13]. Currently, HGS-ETR1/2 mainly relies on inducing apoptosis of tumour cells to exert anti-tumour effects [14–17]. However, it remains unclear whether there exist additional pathways involved in this process. An in-depth understanding of the pattern and mechanism by which HGS-ETR1/2 eliminates tumour cells is helpful to identify novel targets for tumour treatment and lay a foundation for better tumour-killing effects.

Pyroptosis is a newly discovered form of programmed cell death, distinct from the well-established phenomenon of cell apoptosis; cells undergoing pyroptosis form numerous bubble-like protrusions and can swell and expand until rupture, which is a form of lytic death [18, 19]. Pyroptosis is primarily carried out by GSDM-family proteins. It has been reported that in addition to DFNB59, five other GSDM-family proteins, including GSDMA [20, 21], GSDMB [22, 23], GSDMC [24, 25], GSDMD [26, 27] and GSDME [28], can mediate pyroptosis. They all contain a cytotoxic N-terminal domain and a self-inhibiting C-terminal domain. Upon activation through cleavage, the N-terminal domain is released and assembled in the plasma membrane to form pores that destroy the integrity of cell membranes, causing cell rupture and death [29]. As a result, the cell contents, including the inflammatory cytokines IL-1β and IL-18, are released into the extracellular space, causing a strong inflammatory response [30, 31].

Inducing pyroptosis in tumour cells is an important antitumour immune response [32, 33]. DR4/5 is highly expressed on the surface of tumour cells, and its agonist monoclonal antibody HGS-ETR1/2 has a higher utilization rate than TRAIL due to the lack of competitive binding of decoy receptors. In this study, we used hepatocellular carcinoma cells as a research model to explore the killing effect of HGS-ETR1/2 on various hepatocellular carcinoma cells. We found that HGS-ETR1/2 can not only induce apoptosis of cells but also induce GSDME-mediated pyroptosis in hepatoma cells. Further investigation of the mechanism revealed that the carboxypeptidase A4 (CPA4) and AKT signalling pathways can inhibit pyroptosis through positive feedback.

## Results

### HGS-ETR1/2 induces pyroptosis mediated by cleavage of GSDME

**Fig. 1:**
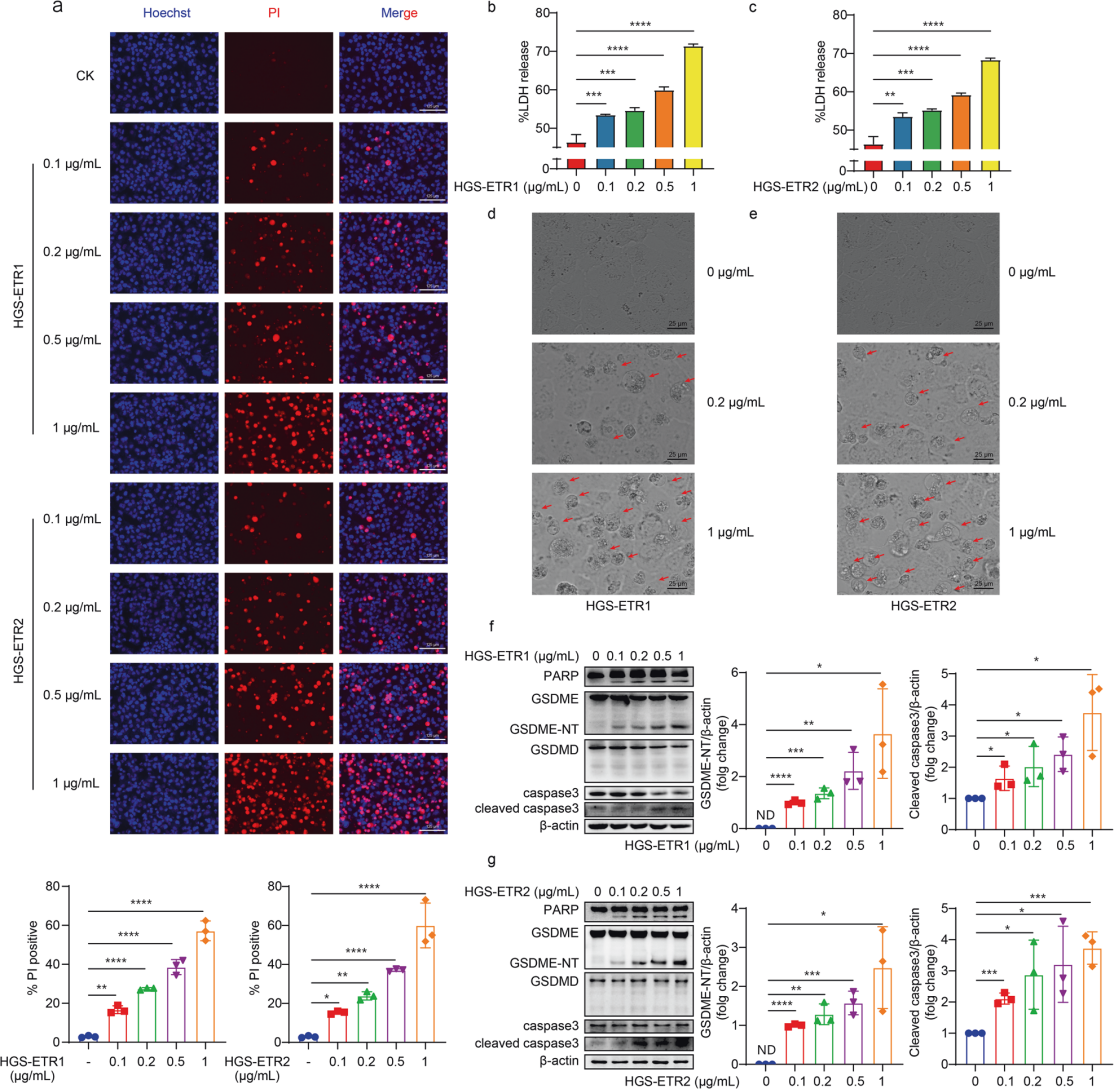
HGS-ETR1/2 induces pyroptosis mediated by cleavage of GSDME. **a** After HLCZ01 cells were treated with different concentrations of HGS-ETR1 (**top**) and HGS-ETR2 (**bottom**) for 8 h, PI/Hoechst stain was added to the cell supernatant, and the cell death was detected by fluorescence microscopy. The PI-positive cells (dead cells) showed red fluorescence. Scale bar, 125 μm. **b, c** After HLCZ01 cells were treated with different concentrations of HGS-ETR1 (**b**) and HGS-ETR2 (**c**) for 8 h, the cell medium was collected and centrifuged, and the supernatant was collected to detect LDH release. %LDH release = sample A490 nm/ positive control A490 nm*100. **d, e** After adding different concentrations of HGS-ETR1 (**d**) and HGS-ETR2 (**e**) to HLCZ01 cells for 8 h, they were photographed by optical microscopy. The red arrows indicate the morphology of the cells when pyroptosis occurred. Scale bar, 25 μm. **f, g** After adding different concentrations of HGS-ETR1 (**f**) and HGS-ETR2 (**g**) to the cells for 8 h, the dead cells suspended in the medium were collected by centrifugation, and the total protein was obtained by lysis together with the adherent cells. The expression levels of PARP, GSDMD, GSDME, caspase-3 and cleaved caspase-3 were detected by western blot, and actin was used as the internal reference protein. Three independent biological replicates were performed for each of the above experiments. One-way ANOVA was used to analyse significant differences (**b, c**) (**p <0.01, ***p <0.001, ****p <0.0001).

In this study, we administered HGS-ETR1/2 to HLCZ01 hepatoma cell. PI/Hoechst staining detection showed that the cells showed a dose-dependent death rate (Fig. 1a), and the release of lactate dehydrogenase (LDH) also showed a dose-dependent increase (Fig. 1b, c), indicating that the cells underwent lysis death. Existing reports have only confirmed that HGS-ETR1/2 can induce cell apoptosis, but other forms of cell death have not been reported. During the examination of cell death morphology by optical microscopy, we observed the presence of swelling, deformation, and bubbles on the surface of cells (Fig. 1d, e), which was consistent with the previously reported pyroptotic morphology. Further detection of GSDM proteins expression in hepatoma cells showed that HGS-ETR1/2 can induce the activation of GSDME, but not GSDMD, by cleavage (Fig. 1f, g). In addition to HLCZ01 cells, we also detected HGS-ETR1/2 induced GSDME cleavage (Fig. S1a, b) and cell lysis death (Fig. S1c, d) in HepG2 and Huh7 hepatoma cells.

### HGS-ETR1/2-induced pyroptosis is regulated by the caspase signalling pathway

**Fig. 2:**
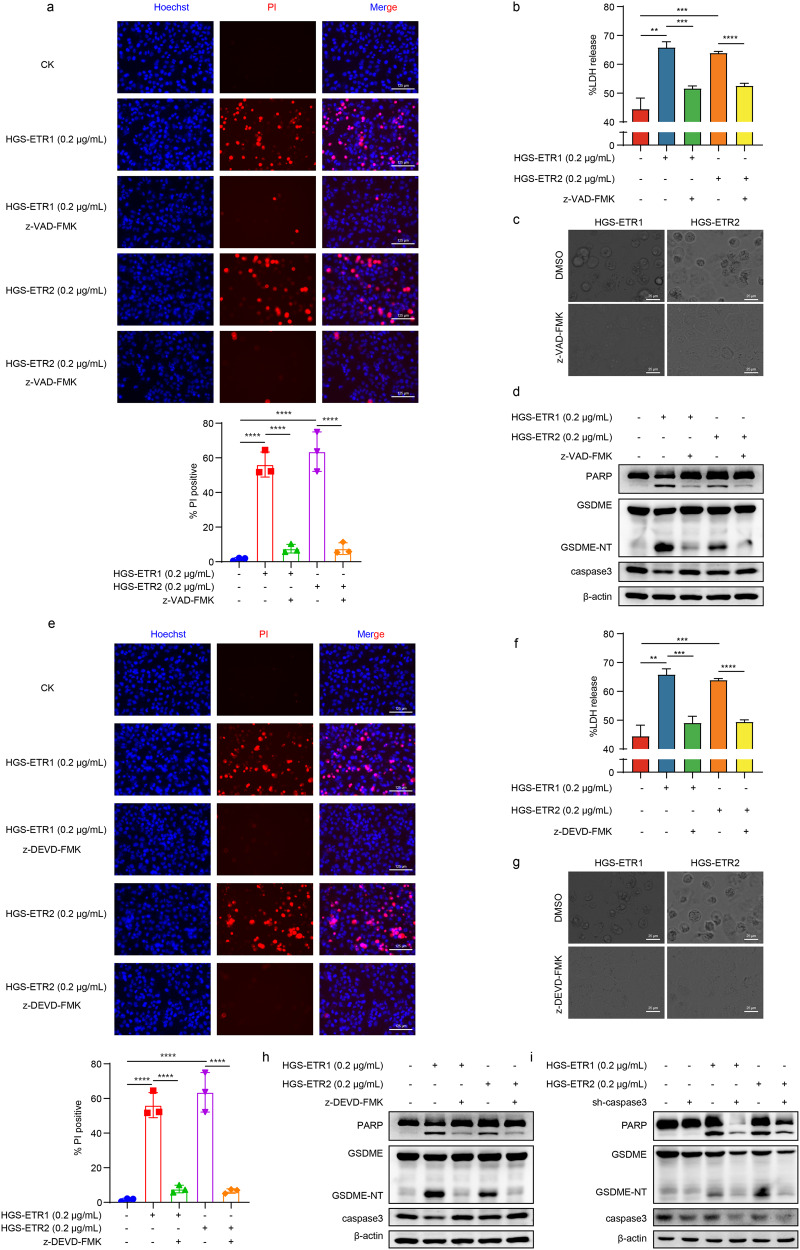
HGS-ETR1/2 induced-pyroptosis is regulated by the caspase signalling pathway. **a, e** A 50 μM concentration of the pan-caspase inhibitor z-VAD-FMK (**a**) or the caspase-3 specific inhibitor z-DEVD-FMK (**e**) was added to the supernatant of HLCZ01 medium. After 1 h, 0.2 μg/mL HGS-ETR1/2 was added, and after 8 h, 1 μL PI and Hoechst dye were added to each well and incubated for 20 min. Cell death was observed by fluorescence microscopy. Scale bar, 125 μm. **b, f** 50 μM z-VAD-FMK (**b**) or z-DEVD-FMK (**f**) was added to the supernatant of HLCZ01 medium, and then HGS-ETR1/2 was added to the medium 1 h later. After 8 h of treatment, the medium was collected and centrifuged, and the supernatant was absorbed to detect the release of LDH. **c, g** 50 μM z-VAD-FMK (**c**) or z-DEVD-FMK (**g**) was added to the supernatant of HLCZ01 medium, and then HGS-ETR1/2 was added to the medium 1 h later, and the pyroptosis morphology was observed by optical microscopy after 8 h. Scale bar, 25 μm. **d, h** 50 μM z-VAD-FMK(**d**) or z-DEVD-FMK(**h**) was added to the supernatant of HLCZ01 medium, and then HGS-ETR1/2 was added to the medium 1 h later. After 8 h, dead cells suspended in the medium were collected by centrifugation, and total proteins were obtained by lysis together with adherent cells. The expression levels of PARP, GSDME and caspase-3 were detected by western blot, and β-actin was used as the internal reference protein. **i** The lentivirus-silenced HLCZ01 cell line with caspase-3 was screened, and proteins were extracted 10 h after the addition of HGS-ETR1/2. The expression levels of PARP, GSDME and caspase-3 were detected by western blot, and β-actin was used as the internal reference protein. Three independent biological replicates were performed for each of the above experiments. One-way ANOVA was used to analyse significant differences (**b, f**) (**p <0.01, ***p <0.001, ****p <0.0001).

To verify whether the pyroptosis induced by HGS-ETR1/2 is regulated by the caspase signal cascade as previously reported, the pan-caspase inhibitor z-VAD-FMK was added to investigate the regulation of pyroptosis; it was found that the number of PI-positive cells decreased (Fig. 2a), LDH release was inhibited (Fig. 2b), the proportion of cells with pyroptotic morphology was reduced (Fig. 2c), and GSDME cleavage was also inhibited (Fig. 2d).

Previous studies have reported that GSDME can be activated by caspase-3 cleavage. To confirm whether HGS-ETR1/2-induced GSDME cleavage is regulated by caspase-3, we added the caspase-3-specific inhibitor z-DEVD-FMK and treated the cells with HGS-ETR1/2. PI/Hoechst staining showed that the number of PI-positive cells decreased (Fig. 2e). The inhibition of pyroptosis was also confirmed by LDH release (Fig. 2f), morphological observation (Fig. 2g) and GSDME cleavage detection (Fig. 2h). In addition, HGS-ETR1/2 induced GSDME cleavage was also detected after caspase-3 was silenced in HLCZ01 and Huh7 cells, confirming that GSDME cleavage-mediated pyroptosis was regulated by caspase-3 (Fig. 2i, S2a, c).

### Screening and transcriptomic analysis of cell lines resistant to HGS-ETR1/2-induced pyroptosis

**Fig. 3:**
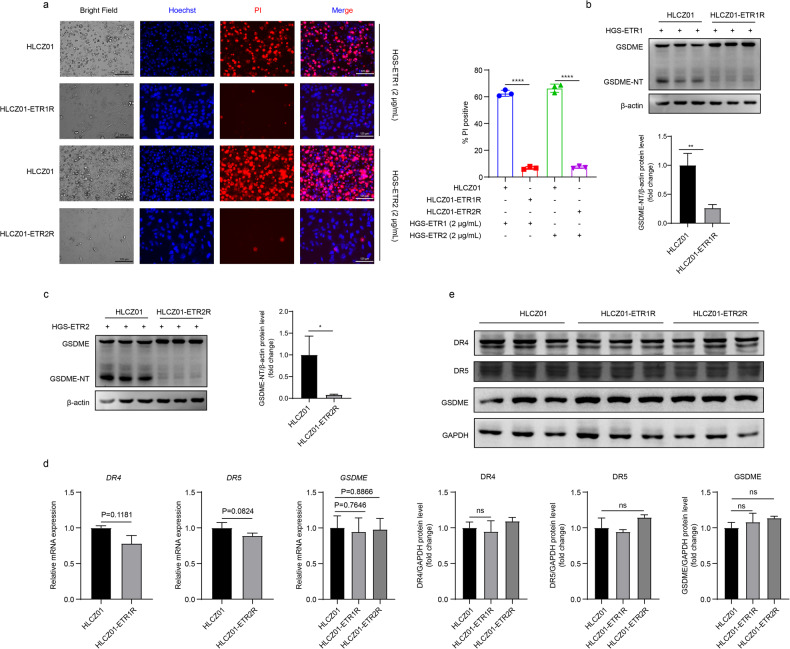
Screening of pyroptosis resistant cell lines induced by HGS-ETR1/2. **a** After screening HLCZ01 cells for drug resistance, 2 μg/mL HGS-ETR1/2 was added to treat HLCZ01 and drug-resistant HLCZ01 cells (HLCZ01-ETR1/2 R) for 8 h, and the pyroptosis of the cells was observed by bright field photography and PI/Hoechst fluorescence photography. Scale bar, 125 μm. **b, c** HLCZ01 and HLCZ01-ETR1/2 R cells were treated with 2 μg/mL HGS-ETR1/2 for 8 h. GSDME cleavage was detected by WB, and β-actin was used as the internal reference protein. **d** The mRNA expression levels of *DR4*, *DR5* and *GSDME* in HLCZ01 and HLCZ01-ETR1/2 R cells were detected by qPCR, with *GAPDH* as the internal reference gene. **e** The protein expression levels of DR4, DR5 and GSDME in HLCZ01 and HLCZ01-ETR1/2 R cells were detected by western blotting, and β-actin was used as the internal reference protein. Three independent biological replicates were performed for each of the above experiments. Two-sided Student’s t test was used to analyse significant differences (**b-e**) (*p <0.05, **p <0.01).

To explore the specific mechanism of HGS-ETR1/2-induced pyroptosis, HLCZ01 cells resistant to HGS-ETR1/2-induced pyroptosis were screened, and the resulting drug-resistant cell lines were named HLCZ01-ETR1R and HLCZ01-ETR2R. The successful screening of drug-resistant cells was confirmed by bright field imaging, PI/Hoechst staining and GSDME cleavage (Fig. 3a–c). Subsequently, by measuring the expression of DR4, DR5 and GSDME, it was determined that the resistance of HGS-ETR1/2 R cells was not caused by differences in the expression of surface receptors or effector molecules (Fig. 3d, e).

**Fig. 4:**
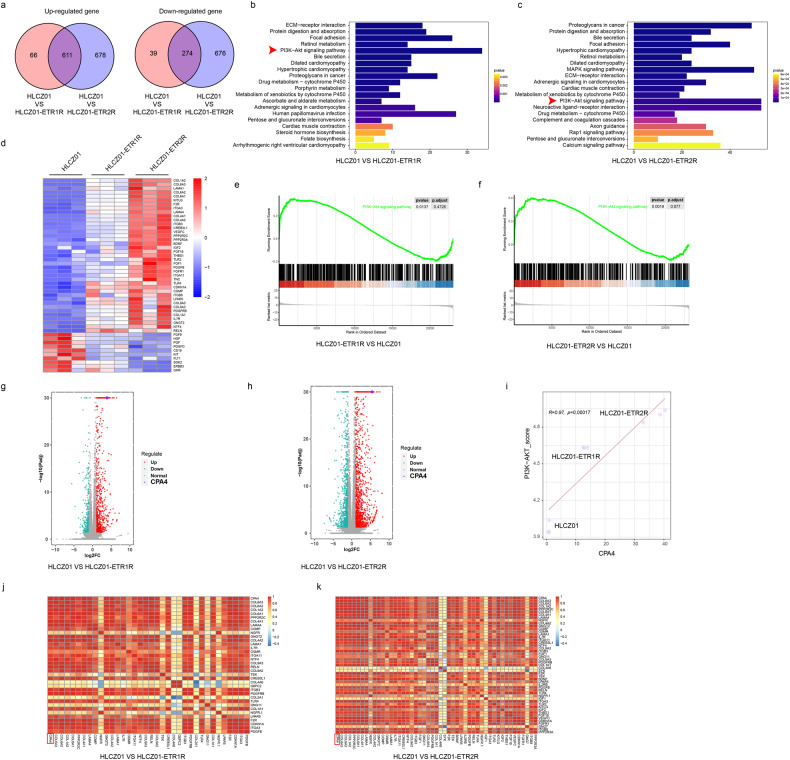
Transcriptomic analysis of cell lines resistant to HGS-ETR1/2-induced pyroptosis. **a** Venn diagram of the differentially expressed genes between HLCZ01-ETR1/2 R and HLCZ01. The left side shows the upregulated genes, and the right side shows the downregulated genes. **b, c** KEGG analysis of the signalling pathway of differential gene enrichment between HLCZ01-ETR1/2 R and HLCZ01, analysis of the number of differential genes in the signalling pathway, and finding that the PI3K-AKT signalling pathway was enriched. **d-f** Heatmap (**d**) and GSEA (**e, f**) analyses were performed on the differentially expressed genes enriched in the AKT signalling pathway in the transcriptome data, and it was confirmed that most genes were mainly upregulated in HLCZ01-ETR1/2 R cells. **g, h** Volcano plot analysis of transcriptome data showed that CPA4 was upregulated in both HLCZ01-ETR1R and HLCZ01-ETR2R. **i-k** Based on transcriptome data, the correlation between the AKT signalling pathway and CPA4 was analysed, and it was confirmed that CPA4 and genes in the AKT signalling pathway were positively correlated in HLCZ01, HLCZ01-ETR1R and HLCZ01-ETR2R cells as a whole (**i**). Correlation heatmap analysis also confirmed that CPA4 was positively correlated with differentially expressed genes in the AKT signalling pathway (**j, k**).

HLCZ01-ETR1/2 R cells were analysed by transcriptional sequencing, and intersection analysis of differentially expressed genes in HLCZ01-ETR1R and HLCZ01-ETR2R showed that 611 genes were upregulated and 274 genes were downregulated (Fig. 4a). A KEGG signalling pathway enrichment analysis showed that the differentially expressed genes of the two drug-resistant cell lines were enriched in the PI3K-AKT signalling pathway (Fig. 4b, c). Further analysis showed that the enriched AKT signalling pathway genes were upregulated in both drug-resistant cell lines, indicating that the activity of the AKT signalling pathway was increased in drug-resistant cells (Fig. 4d–f). At the same time, it was also found that the *CPA4* gene, which has been reported to be able to positively regulate the AKT signalling pathway, was significantly upregulated in both drug-resistant cell lines (Fig. 4g, h). We also analysed the relationship between the AKT signalling pathway and CPA4 using transcriptomic data, and confirmed the positive correlation between the two, but further research is needed to explore their relationship with pyroptosis (Fig. 4i–k).

### The AKT pathway regulates HGS-ETR1/2-induced pyroptosis

**Fig. 5:**
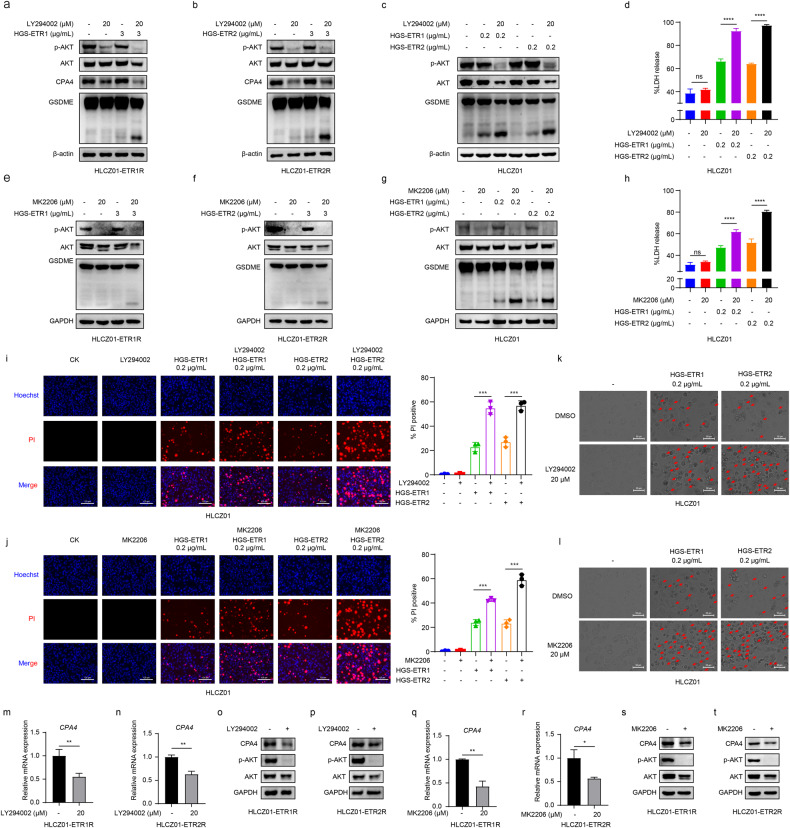
The AKT pathway regulates HGS-ETR1/2-induced pyroptosis. **a-c** The AKT inhibitor LY294002 (20 μM) was added to HLCZ01-ETR1/2 R (**a,** **b**) or HLCZ01(**c**), and the cells were treated with HGS-ETR1/2 1 h later. Proteins were collected 8 h later. The expression of AKT, p-AK and GSDME was detected by western blotting, and β-actin was used as the internal reference protein. **d, h** HLCZ01 cells were treated with 20 μM LY294002 (**d**) or MK2206 (**h**) for 1 h, and HGS-ETR1/2 was added for 8 h. After the treatment, the cell supernatant was collected and LDH release in the supernatant was detected by the kit. **e-g** The AKT inhibitor MK2206 (20 μM) was added to HLCZ01-ETR1/2 R (**e, f**) or HLCZ01(**g**), and the cells were treated with HGS-ETR1/2 1 h later. Proteins were collected 8 h later. The expression of AKT, p-AK and GSDME was detected by western blotting, and β-actin was used as the internal reference protein. **i, j** HLCZ01 cells were treated with LY294002 (**i**) or MK2206 (**j**) for 1 h, followed by HGS-ETR1/2 for 8 h, staining with PI and Hoechst, and fluorescence photography. Scale bar, 125 μm. **k, l** 20 μM LY294006 (**k**) or MK2206(**l**) was added to the supernatant of HLCZ01 medium, and then HGS-ETR1/2 was added to the medium 1 h later, and the pyroptosis morphology was observed by optical microscopy after 8 h. Scale bar, 50 μm. **m, n, q, r** 20 μM LY294006 (**m, n**) or MK2206 (**q, r**) was added to HLCZ01-ETR1/2 R, and RNA was collected 12 h later. The expression of the *CPA4* gene was detected by qPCR, and *GAPDH* was used as the internal reference gene. **o, p, s, t** 20 μM LY294006 (**o, p**) or MK2206 (**s, t**) was added to HLCZ01-ETR1/2 R, and protein was collected 12 h later. The expression of CPA4 was detected by western blotting and GAPDH was used as the internal reference protein. Three independent biological replicates were performed for each of the above experiments. Two-sided Student’s t test was used to test for significant differences (*p <0.05, **p <0.01, *** *p* <0.001, **** *p* <0.0001).

To investigate the role of AKT signalling pathway activation in HGS-ETR1/2-induced pyroptosis, the enhanced activation of p-AKT in HLCZ01-ETR1/2 R cells was first verified through western blot analysis (Fig. S3a). Subsequently, the AKT signalling pathway inhibitors LY294002 or MK2206 were added to HLCZ01-ETR1/2 R, followed by HGS-ETR1/2 treatment of cells. The results showed that after LY294002 and MK2206 inhibited AKT phosphorylation, HGS-ETR1/2-induced GSDME cleavage was enhanced (Fig. 5a, b, e, f). Similarly, we added LY294002 and MK2206 to sensitive HLCZ01 cells for treatment, and found that HGS-ETR1/2-induced GSDME cleavage was enhanced after AKT phosphorylation was inhibited (Fig. 5c, g). Meanwhile, the release of LDH, PI-positive and pyroptotic morphological cells also increased (Fig. 5d, h, i, j, k, l). The above experiments were carried out in the hepatocellular carcinoma cells HepG2 and Huh7, and similar results were obtained: AKT inhibitors can promote the induction of pyroptosis by HGS-ETR1/2 (Fig. S3b, h). These results indirectly indicate that the AKT signalling pathway can inhibit HGS-ETR1/2-induced pyroptosis.

To define the relationship between the AKT signaling pathway and CPA4 in HLCZ01-ETR1/2 R cells, the expression of CPA4 was detected by qPCR and western blot after adding LY294002 and MK2206, and it was found that the transcriptional level and protein expression of CPA4 are decreased after being treated with LY294002 and MK2206 (Fig. 5m–t). These results indicate that the AKT signaling pathway can positively regulate CPA4 expression.

### Carboxypeptidase A4 (CPA4) regulates HGS-ETR1/2-induced pyroptosis

**Fig. 6:**
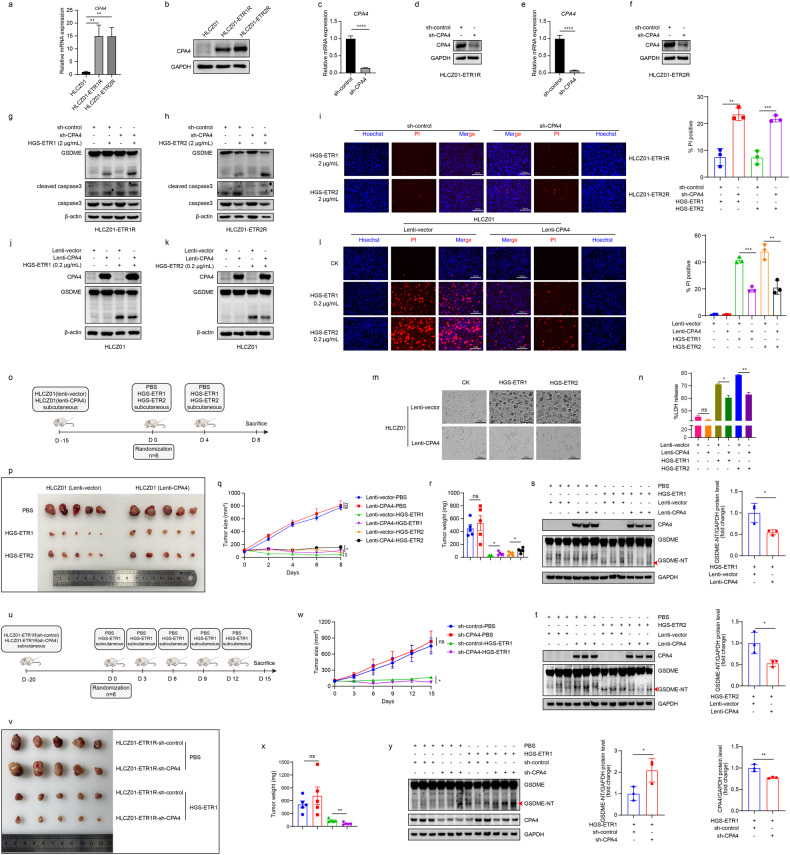
Carboxypeptidase A4 (CPA4) regulates HGS-ETR1/2-induced pyroptosis. **a, b** The upregulated expression of CPA4 in HLCZ01-ETR1/2 R cells was verified by qPCR (**a**) and western blotting (**b**). **c-f** Lentiviral sh-CPA4 supernatant was added to HLCZ01-ETR1/2 R cell culture medium, stable cell lines were screened with puromycin, and the silencing effect was detected by qPCR (**c, e**) and western blotting (**d, f**). **g, h** CPA4-silenced HLCZ01-ETR1R (**g**) and HLCZ01-ETR2R (**h**) cells were treated with 2 μg/mL HGS-ETR1 or HGS-ETR2 for 10 h, and the protein expression of GSDME, caspase-3, and cleaved caspase-3 was detected by western blotting, β-actin was used as the internal reference protein. **j, k** Lenti-CPA4 supernatant was added to HLCZ01 cells, CPA4 was overexpressed, and stable cell lines were screened with puromycin. After 10 h of addition of HGS-ETR1/2, proteins were collected to detect the expression of CPA4 and the cleavage of GSDME. β-actin was used as the internal reference protein. **i, l** HGS-ETR1/2 was added to HLCZ01-ETR1/2 R cells that silenced CPA4 (**i**) and HLCZ01 cells that overexpressed CPA4 (**l**). Ten hours later, PI and Hoechst dye were added to the cell supernatant for staining, and the cell death was recorded by fluorescence photography. Scale bar, 300 μm (**i**) or 125 μm (**l**). **m** HGS-ETR1/2 was added to HLCZ01 cells that overexpressed CPA4, and the pyroptosis morphology of the cells was observed 10 h later by bright field photography. **n** HGS-ETR1/2 was added to HLCZ01 cells that overexpressed CPA4, and the supernatant was absorbed 10 h later to detect the release of LDH in the supernatant. **o-t** Schematic diagram of NCG mice treated with HLCZ01(lentiviral vector/CPA4) injection (**o**). After the back hair removal of mice, 5 million HLCZ01 cells overexpressing CPA4 or vector were injected into them. When the tumour grew out, its volume was measured and recorded every 2 days. When the tumour volume was approximately 100 mm^3^, the mice were randomly grouped and injected with HGS-ETR1/2 or PBS (10 mg/kg), and then injected again 4 days later. After the second injection, the mice were sacrificed 4 days later, and the tumours were removed for photographic observation (**p**) and weighed. According to the measured tumour volume, growth curve analysis was performed for tumours in different groups (**q**). The tumour was removed and weighed to analyse the difference in tumour weight between different groups of mice (**r**). In order to have the samples on the same PVDF membrane, three tumor tissues were randomly selected from each group to extract proteins. The expression of GSDME and CPA4 in the tumor tissues was detected by western blot (**s, t**). **u-y** Schematic diagram of NCG mice injected with HLCZ01-ETR1R(sh-control/CPA4) (**u**). After the back hair removal of mice, 5 million silenced CPA4 or control HLCZ01-ETR1R cells were injected, and the volume was measured every 3 days after the growth of the tumour, and the volume was measured and recorded. When the tumour volume was approximately 100 mm^3^, the mice were randomly grouped and injected with HGS-ETR1 (10 mg/kg) or PBS. After 3 days, the mice were injected again for a total of 5 times. After the fifth injection, the mice were sacrificed 3 days later, and the tumours were removed for photo observation (**v**) and weighed. Growth curve analysis was performed for different groups of tumours based on the measured tumour volume (**w**). Tumour weight differences between different groups of mice were analysed by weighing the removed tumours (**x**). Three tumor tissues were randomly selected from each group to extract proteins, and the expression of GSDME and CPA4 in the tumor tissues was detected by western blot (**y**). The experiment was independently repeated two (**o-t**) or three times(**a-n**). Two-sided Student’s t test was used to analyse significant differences. (*p <0.05, **p <0.01, *** *p* <0.001, **** *p* <0.0001).

To further explore the regulatory mechanism of CPA4 in pyroptosis, we first confirmed that CPA4 was upregulated in both HLCZ01-ETR1R and HLCZ01-ETR2R cells by qPCR and western blotting (Fig. 6a, b). Subsequently, HLCZ01-ETR1/2 R cells with *CPA4* gene silencing were treated with HGS-ETR1/2 (Fig. 6c–f). The immunoblotting results showed that GSDME cleavage was enhanced in CPA4-silenced cells (Fig. 6g, h), and the number of PI-positive cells also increased (Fig. 6i). Overexpression of CPA4 in HLCZ01 cells via a lentiviral infection system was found to inhibit HGS-ETR1/2-induced GSDME cleavage (Fig. 6j, k), and the number of pyroptotic cells decreased (Fig. 6m), LDH release (Fig. 6n) and the number of PI-positive cells decreased (Fig. 6l).

Subcutaneous tumour transplantation was performed in NCG mice (Fig. 6o), and the results showed that overexpression of CPA4 in HLZ01 cells could resist the tumour-killing effect of HGS-ETR1/2 (Fig. 6p). After subcutaneous injection of HGS-ETR1/2, the mice in the overexpression group had significantly greater tumour weight and volume than those in the control group (Fig. 6q, r). The detection of GSDME protein in mouse tumour by western blot also found that the group with overexpression of CPA4 could inhibit the occurrence of pyroptosis in tumor tissues compared with the control group (Fig. 6s, t). However, after CPA4 was stably silenced in HLCZ01-ETR1R, subcutaneous tumours were implanted in NCG mice, and when the tumour volume was approximately 100 m^3^, subcutaneous injection of HGS-ETR1 was performed to kill the tumour (Fig. 6u). Compared with the control group, the silencing CPA4 group could promote the killing effect of HGS-ETR1 on tumours in mice (Fig. 6v), and the tumour volume and weight were smaller than those of the control group (Fig. 6w, x). Simultaneously, GSDME protein cleavage was stronger in the tissues of the silenced CPA4 group than the control group (Fig. 6y). The above results confirmed the negative regulatory effect of CPA4 on HGS-ETR1/2-induced pyroptosis at both the cellular and organismal levels.

### CPA4 regulates HGS-ETR1/2- inducedpyroptosis by promoting AKT phosphorylation

**Fig. 7:**
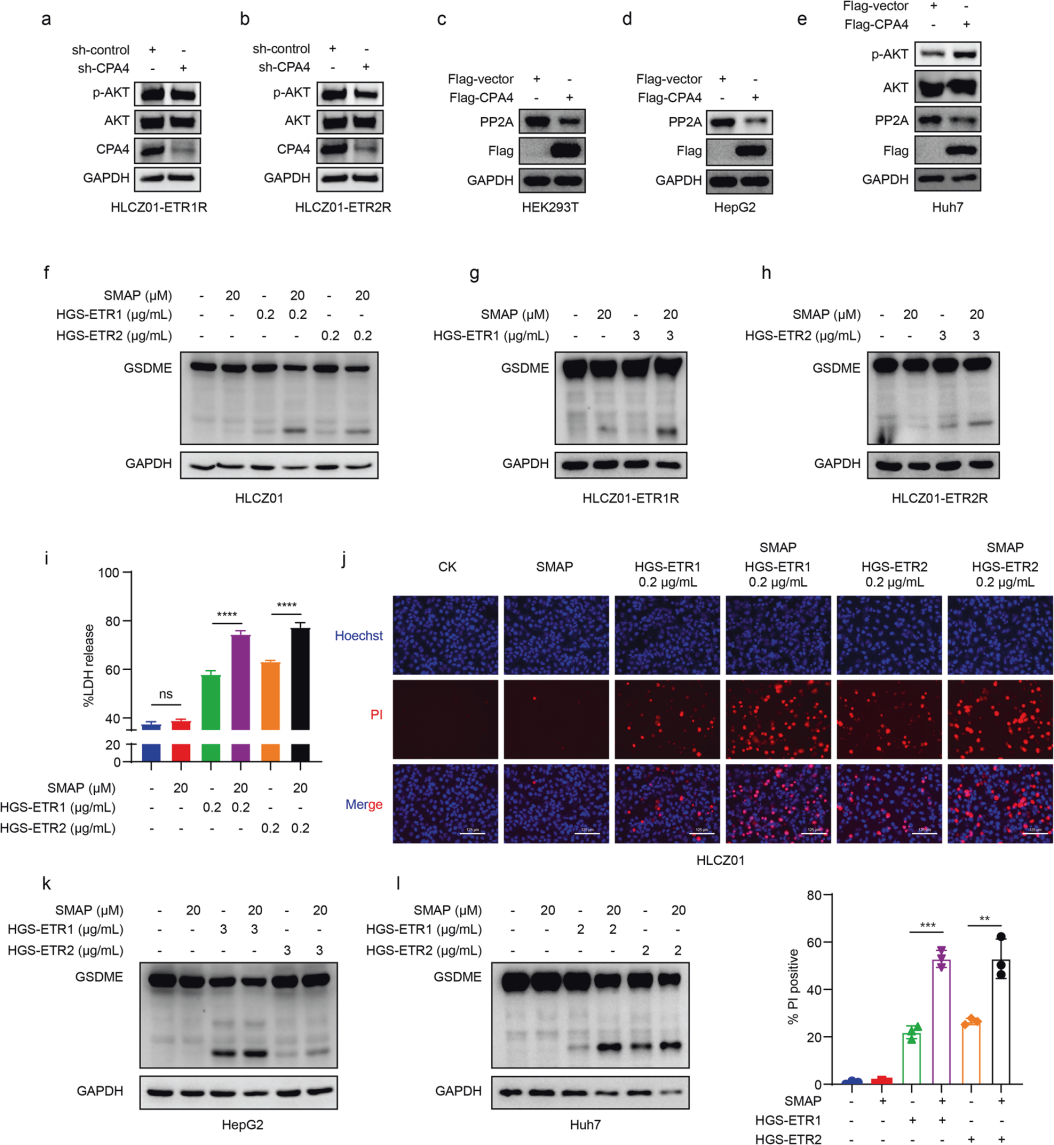
CPA4 regulates HGS-ETR1/2 induced pyroptosis by promoting AKT phosphorylation. **a, b** The expression of p-AKT and AKT was detected in HGS-ETR1 (**a**) and HGS-ETR2 (**b**) cell lines silenced by CPA4, and GAPDH was used as the internal reference protein. **c-e** After flag-CPA4 was overexpressed in HEK293T (**c**), HepG2 (**d**) and Huh7 (**e**) cells, AKT phosphatase PP2A protein expression was detected, and GAPDH was used as the internal reference protein. **f-h** The PP2A activator SMAP (20 μM) was first added to HLCZ01 (**f**), HLCZ01-ETR1R (**g**) and HLCZ01-ETR2R (**h**), and the treatment was followed by the addition of HGS-ETR1 or HGS-ETR2 1 h later. The protein was collected 8 h later to detect the cleavage of GSDME, and GAPDH was used as the internal reference protein**. i** After 20 μM SMAP was added to HLCZ01 for 1 h, 0.2 μg/mL HGS-ETR1/2 was added to HLCZ01 for 8 h. The cell supernatant was collected and LDH release in the supernatant was detected. **j** HLCZ01 cells were treated with 20 μM SMAP for 1 h, followed by 0.2 μg/mL HGS-ETR1/2. After 8 h of treatment, PI and Hoechst dye solution were added to the medium and incubated for 20 min. Cell death was detected by fluorescence photography. Scale bar, 125 μm. **k, l** HepG2 (**k**) and Huh7 (**l**) cells were treated with 20 μM SMAP for 1 h, and then HGS-ETR1/2 for 8 h. Proteins were collected and GSDME cleavage was detected. GAPDH was used as the internal reference protein. The experiment was independently replicated three times. A two-sided Student’s t test was used to analyse significant differences. (***p <0.001).

To further explore the relationship between CPA4 and the AKT signalling pathway, we measured the phosphorylation of AKT in HLCZ01-ETR1/2 R cells with silenced CPA4, and found that the phosphorylation level of AKT was decreased after the silencing of CPA4 (Fig. 7a, b). We ruled out a direct interaction between the two by co-immunoprecipitation experiments in a variety of cells (Fig. S3a–d). It has been reported that K63-linked ubiquitination of AKT can affect its phosphorylation [34, 35]. However, overexpression of CPA4 in different cells did not affect K63-linked ubiquitination of AKT (Fig. S4e-g). We further explored the dephosphorylation process and found that CPA4 downregulated the AKT phosphatase PP2A, suggesting that CPA4 may inhibit the dephosphorylation process by downregulating its phosphatase, thus increasing the percentage of phosphorylation (Fig. 7c–e). After the addition of the PP2A activator SMAP to a variety of cells, HGS-ETR1/2- inducedpyroptosis was promoted, GSDME cleavage was enhanced (Fig. 7f–h, k, l), LDH release was increased (Fig. 7i), and the number of PI-positive cells was increased (Fig. 7j), indicating that activation of PP2A can positively regulate pyroptosis. These results suggest that CPA4 may enhance AKT phosphorylation levels by inhibiting the AKT dephosphorylation process, thereby negatively regulating pyroptosis.

### CPA4 can regulate pyroptosis independently of its carboxypeptidase activity

CPA4 belongs to the carboxypeptidase A family and is a metallopeptidase. Its activity depends on its binding to zinc ions [36, 37]. According to the structural analysis report [38], we mutated the three amino acid sites where it binds to zinc ions to investigate whether its pyroptosis-regulating function depends on its carboxypeptidase activity (Fig. S5a). HGS-ETR1/2 was added to the HLCZ01 stable cell line of lentivirus-infected CPA4-mutant. After the zinc-binding site of CPA4 was mutated, the downregulation of LDH release (Fig. S5b), the inhibition of regulatory pyroptosis (Fig. S5c), the reduction of PI-positive cells (Fig. S5d) and the inhibition of GSDME protein cleavage (Fig. S5e) were not affected, suggesting that the regulation of pyroptosis by CPA4 is independent of its metal carboxypeptidase activity.

### CPA4 is associated with poor tumour prognosis in the clinic

To explore the clinical significance of CPA4, we purchased tissue microarrays (Shanghai OUTDO BIOTECH Co., Ltd.), and performed immunohistochemical staining on them [39]. The staining was scored based on its depth and area of staining [40] (Fig. S6a), and the results were analysed in combination with clinical information. Our findings showed that the expression of CPA4 protein in HCC tissues was significantly higher than that in adjacent tissues (Fig. S6b). Further survival curve analysis showed that the high expression of CPA4 was associated with poor prognosis of HCC patients (Fig. S6c). Western blot analysis of HCC tissue from patients found that in patients 1, 2, 3, 4, and 6, the expression of CPA4 protein was positively correlated with p-AKT, while it was negatively correlated with PP2A expression and GSDME cleavage, which is consistent with the cellular research in this study (Fig. S6d). According to an analysis using the Kaplan‒Meier Plotter database (http://kmplot.com/analysis/index.php?p=background), high CPA4 expression is associated with poor prognosis in a variety of cancers except liver cancer (Fig. S6e, m). The high expression of CPA4 in various cancer tissues suggests that CPA4 could be used as a potential target for the clinical treatment of tumours.

## Discussion

Pyroptosis is a type of cell death that has gained intensive attention in recent years. This process is mediated by the GSDM-family of proteins, which undergo cleavage leading to the accumulation of N-terminal peptides and subsequent formation of membrane holes, resulting in cell rupture, release of the cellular contents, and ultimately cell death by lysis [18]. Pyroptosis is a type of inflammatory cell death. Induction of tumour pyroptosis is an important means of antitumour immunity, which can inhibit tumour progression by altering the tumour microenvironment [41].

In this study, through morphology, fluorescence double staining, LDH release and western blotting, we ascertained for the first time that the humanized agonist antibody HGS-ETR1/2, targeting DR4/5, can induce pyroptosis rather than just apoptosis as previously reported. We also demonstrated that two pyroptotic executive proteins expressed in HCC cells, only GSDME was cleaved and GSDMD was not (Fig. 1 and Fig. S1). The reason for this may be that the caspase cascade of the death receptor signalling pathway activates caspase-3, which can cleave GSDME, rather than caspase-1, 4 and 5, which can cleave GSDMD [28, 42].

HGS-ETR1/2 is similar to TRAIL and can bind to DR4/5 on the cell surface [43]. However, unlike TRAIL, HGS-ETR1/2 cannot bind to other receptors that do not transmit death signals, making it more efficient than TRAIL in inducing cell death. TRAIL or HGS-ETR1/2 can kill multiple tumour cells by activating the caspase cascade signalling pathway, but the only reports on their killing methods are focused on apoptosis [44]. The study of the induction of other forms of cell death can help better regulate the tumour immune process.

CPA4 has been previously reported to promote epithelial-mesenchymal transition in pancreatic cancer [45], cardiomyocyte hypertrophy and the growth of non-small cell lung cancer through positive regulation of the AKT signalling pathway [40, 46–48], and it is closely related to the development of a variety of cancers. There are also a few reports that CPA4 can inhibit the apoptosis process of tumour cells through the AKT signalling pathway [49], but no studies have explored how CPA4 regulates the phosphorylation process of AKT, and no studies on its relationship with pyroptosis have been reported. AKT plays an important role in promoting tumour cell proliferation and inhibiting apoptosis [50–52], but its regulation of pyroptosis has rarely been reported.

**Fig. 8:**
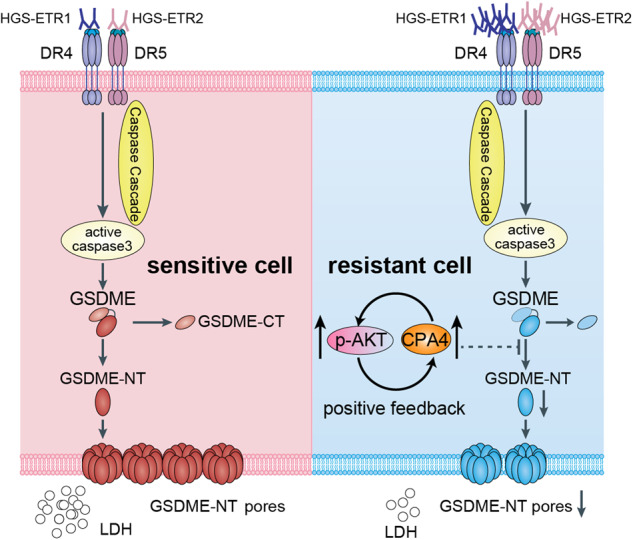
Schematic diagram of CPA4 and p-AKT synergistically negatively regulating GSDME-mediated pyroptosis. When the tumour drug HGS-ETR1/2 induces pyroptosis through the death receptor signalling pathway, CPA4 and p-AKT can synergistically inhibit the cleavage of GSDME through positive feedback, thereby inhibiting pyroptosis.

In this study, transcriptomic analysis revealed the CPA4 and AKT signalling pathways were upregulated in pyroptosis-resistant cells (Fig. 4), both of which were confirmed to negatively regulate pyroptosis in subsequent experiments (Figs. 5, 6). When exploring the relationship between the two, we found that CPA4 can promote AKT phosphorylation by inhibiting the expression of the AKT phosphatase PP2A (Fig. 7), and then inhibit pyroptosis by positively regulating AKT phosphorylation; we also found that after AKT phosphorylation is inhibited, the transcription of CPA4 is reduced (Fig. 5m–t), thereby decreasing CPA4 expression and thus promoting pyroptosis (Fig. 8). Meanwhile, this study also found that CPA4 is clinically associated with poor prognosis in various tumours, and in liver cancer tissue, the expression of CPA4 protein is negatively correlated with the expression of PP2A and the cleavage of GSDME, while it is positively correlated with the expression of p-AKT (Fig. S6). However, this study needs further clarification of how CPA4 affects the expression of the AKT phosphatase PP2A and how AKT signalling affects the transcription of CPA4. Overall, the results of this study have identified new research targets and mechanisms for pyroptosis, and laid a good foundation for subsequent research on anti-tumour immunity.

## Materials And Methods

### Plasmids

Plasmids containing V5-AKT, HA-ub, HA-K48 and HA-K63 were all stored in our laboratory. The construction of flag-CPA4 and lenti-CPA4 recombinant plasmids was based on the cDNA of HLCZ01-ETR2R as the template, and the primers (Table S1) were designed and amplified with PCR reagents from the high-fidelity PCR kit KOD Plus-Neo (TOYOBO). After enzyme digestion, the product was recovered and ligated into the p3 × Flag-CMV-14 vector (Sigma-Aldrich) or pCDH-CMV-MCS-EF1-GFP vector (System Biosciences). After transformation into *Escherichia coli Trans1-T1* (TransGen Biotech), select monoclonal clones were selected and sent to Sangon Biotech (Shanghai) for sequencing. After the sequencing was correct, the plasmid was extracted. The mutant plasmid of flag-CPA4 was constructed by the Mut Express II Fast Mutagenesis Kit V2 (Vazyme). For the construction of sh-caspase-3 and sh-CPA4 lentivirus plasmids, primers (target sequence of sh-caspase-3 (5’ → 3’): GTGGAATTGATGCGTGATGTT; sh-CPA4 (5’ → 3’): GAGCAGTAATAACTTCAACTA) were synthesized for their gene target sequences. The two primers were annealed and recovered, and then the recovered products were connected to the pGreenPuro vector (System Biosciences) and transformed. Monoclonal clones were selected to confirm correct sequencing, and the plasmids were extracted.

### Cells

The human hepatic cell line HLCZ01 was isolated and stored in our laboratory. Huh7 and HepG2 cells were purchased from the American Type Culture Collection (ATCC). HLCZ01 cells were cultured on collagen-coated plates containing Dulbecco’s Modified Eagle Medium Nutrient Mixture F-12 (DMEM/F-12) supplemented with 10% (v/v) foetal bovine serum (FBS) (Gibco), 40 ng/mL of dexamethasone (Sigma), 1 × insulin-transferrin-selenium (ITS) (Lonza), and 1% penicillin-streptomycin (Thermo Fisher Scientific). Other cells were cultured in Dulbecco’s Modified Eagle Medium (DMEM) supplemented with 10% FBS, nonessential amino acids, and 1% penicillin-streptomycin. All cells were grown at 37°C in a 5% CO_2_ incubator (Thermo Fisher Scientific).

### Antibodies and reagents

The antibodies used in this study were as follows: PARP (9532 S), caspase-3 (9662 S), cleaved caspase-3 (9661 T), AKT (4691 S) and phospho-AKT (4060 S), which were purchased from Cell Signal Technology. GSDME (ab215191), GSDMD (ab210070) and PP2A (ab32104) were purchased from Abcam. DR4 (1139) and DR5 (2019) were purchased from ProSci. CPA4 (26824-1-AP) was purchased from Proteintech. HA (923501) was purchased from BioLegend. GAPDH (MAB374)), goat anti-mouse IgG (HRP-linked) (AP124P) and goat anti rabbit IgG (HRP-linked) (AP132P) were purchased from Merck Millipore. Flag (F3165) and β-actin (A5441) were purchased from Sigma-Aldrich. V5 (R960-25) was purchased from Thermo Fisher Scientific. Normal mouse IgG (sc-45123) was purchased from SANTA CRUZ. Both HGS-ETR1 and HGS-ETR2 were gifted by Human Genome Sciences.

The other reagents used in this study were as follows: Thermo Scientific SuperSignal West Pico PLUS (34580) and IP lysis buffer (87787) were purchased from Thermo Fisher Scientific. LY294002 (9901) was purchased from Cell Signal Technology. SMAP (S8774), z-VAD-FMK (S7023) and z-DEVD-FMK (S7312) were purchased from Selleck. MK2206 (SF2712) was purchased from Beyotime Biotechnology. A protease inhibitor cocktail (C0001) and a phosphatase inhibitor cocktail (C0002) were purchased from TargetMol. RIPA lysis buffer (PH0316) was purchased from PHYGENE.

### Mice

Male NCG mice aged 5-6 weeks were purchased from Nanjing GemPharmatech Co., Ltd., and raised under specific-pathogen-free conditions at Hunan University.

For the CPA4 overexpression experiment, 5 × 10^6^ HLCZ01 (lentiviral vector/CPA4) cells were injected subcutaneously into the right side of the back of mice after the hair was removed from the area. Tumour dimensions were measured with vernier callipers every 2 days, and volume was calculated as follows: tumour volume (mm^3^) = length × width^2 ^× 0.5. When the tumour size reached approximately 100 mm^3^, mice injected with the two cell lines were randomly divided into three groups with 6 mice per group. HGS-ETR1/2 was injected subcutaneously next to the tumour at a dose of 10 mg/kg, PBS was injected as a control, and then injected again 3 days later. The anaesthetized mice were sacrificed on the 8th day after the injection, and the tumours were removed and weighed.

For the CPA4 silencing experiment, 5 × 10^6^ HLCZ01 (sh-control/CPA4) cells were injected subcutaneously into the right back of mice after skin hair removal. Tumour dimensions were measured with vernier callipers every 3 days to calculate volume. When the tumour volume reached approximately 100 mm^3^, mice injected with the two cell lines were randomly divided into two groups with 6 mice per group. HGS-ETR1 was injected subcutaneously next to the tumour at a dose of 10 mg/kg, PBS was injected as a control, and the injection was repeated every 3 days for a total of 5 injections. On the 15th day after injection of the HGS-ETR1 or PBS, the mice were sacrificed, and the tumour were removed and weighed.

All experiments were conducted in strict accordance with the relevant requirements of laboratory animal ethics.

### Patient samples and ethical regulation

The patient tissue samples involved in this study were obtained from Hunan Cancer Hospital (Changsha, China) with the patient’s knowledge and consent, and approved by the institutional review board of Hunan Cancer Hospital.

### LDH release assay

Cell lysis was determined by measuring the activity of lactate dehydrogenase (LDH) released into the medium using the CytoTox 96 Non-Radioactive Cytotoxicity Assay (Promega) according to the manufacturer’s instructions.

### Hoechst 33342/PI double fluorescence staining

Cell death was analysed by Hoechst 33342 and PI staining. HLCZ01 cells were seeded into 12-well plates. When the cells converged to approximately 50% confluence in the bottom area of the Petri dish, the medium was replaced with fresh medium, and HGS-ETR1/2 was added. After 10 h, 1 μL of PI Hoechst 33342 (Solarbio, China) was added simultaneously into each well for 20 min. The cells were photographed using a fluorescence microscope. The PI-positive cells were quantitatively analyzed by ImageJ software in three different fields.

### RNA extraction and quantitative real-time PCR (qRT-PCR)

The total RNA of cells was extracted by the TRIzol reagent (Invitrogen, USA), and cDNA was generated using the HiScript Q RT SuperMix for qPCR (+gDNA wiper) (Vazyme, China) according to the manufacturer’s instructions. The cDNA was used as a template for qRT-PCR analysis. qRT-PCR was performed using the SYBR Green Premix Pro Taq HS qPCR Kit (accurate biology, China) on a Mastercycler ep realplex (Eppendorf, Germany). The housekeeping gene GAPDH was used as an internal reference gene. The relative expression content was calculated by the relative quantitative method (2 − ΔΔCt). The primers are presented in Table S2.

### Protein preparation and western blotting

The medium was discarded, and the cells were washed twice with PBS. RIPA buffer containing a protease inhibitor cocktail was added, and the supernatant was put on ice for 30 min and centrifuged at 12000 rpm at 4 °C for 15 min. The supernatant protein was collected and the sample was prepared after quantification by BCA.

For the cells treated with HGS-ETR1/2, the cell supernatant was centrifuged and dead cells were collected. After the adherent cells were washed twice with PBS, RIPA buffer containing a protease cocktail was added to the cell sample for lysis on ice and transferred to a tube containing cell precipitation. Supernatant protein was obtained by centrifugation and BCA quantification was performed before sample preparation.

After the protein samples were separated by SDS-PAGE, they were transferred to PVDF membranes, blocked with TBST containing 5% skim milk for 2 h at room temperature, incubated at 4 °C with the primary antibody overnight, washed three times with TBST, replaced with the HRP-labelled secondary antibody, and incubated at room temperature for 2 h. After TBST was washed for three times, horseradish peroxidase (HRP) chemiluminescent substrate was added for signal detection and a chemiluminescence imaging system was used to obtain photographs.

### Lentiviral production and infection

For a 100 mm cell culture dish, 8 μg constructed lentiviral recombinant plasmid, 8 μg psPAX2 plasmid and 2.7 μg pMD2.G plasmid were added to an EP tube containing 400 μL OPTI. At the same time, 36 μL lipo2000 (Thermo Fisher Scientific, USA) transfection reagent was added into another EP tube containing 400 μL OPTI. After the two tubes were incubated at room temperature for 5 min, they were gently mixed and incubated at room temperature for 20 min, and the total system was added into the petri dish. After transfection for 24 h, the culture medium was changed, and the lentivirus in the cell supernatant was collected and filtered through a 0.45 μm filter membrane (Merck Millipore) after another 12 h. The filtered supernatant was used to infect cells and stable cell lines were screened with puromycin (Thermo Fisher Scientific, USA).

### Screening and transcriptomic analysis of HGS-ETR1/2 resistant cells

HGS-ETR1/2 was added to HLCZ01 cell medium in a concentration gradient way to induce drug-resistant cell lines. First, when HLCZ01 cells were growing well, HGS-ETR1/2 at a concentration of 0.2 μg/mL was added to the medium. The dead cells in the supernatant were discarded and the living cells were cultured by subculture. After the cells could grew stably at this concentration, the concentration of HGS-ETR1/2 was increased to 0.5 μg/mL. After the cells were stabilized, the added concentration of HGS-ETR1/2 was increased by the same method. When the addition concentration of HGS-ETR1/2 was increased to 2 μg/mL, the drug resistance of HLCZ01 cells that could grow continuously and stably at this concentration was verified.

### Co-immunoprecipitation

Forty-eight hours after transfection, the cells were washed twice with cold PBS, lysed on ice with IP buffer containing protease inhibitor cocktail (TargetMol, USA) for 30 min, and the fully lysed cells were transferred to EP tubes and centrifuged at 13500 rpm for 15 min at 4 °C, the supernatant was collected, and 10 μL was absorbed for sample preparation as input. The remaining proteins were incubated with primary antibody at 4 °C overnight, and protein G agarose (Millipore, USA) washed 5 times with PBS was added, incubated at 4 °C for 6 h, and then washed 5 times with PBS. Finally, 40 μL 2 × loading buffer was added to prepare the sample, which was used for western blot analysis.

### Statistical analysis

The data were processed and analysed with GraphPad Prism 8.0.2. The data are presented as the means ± S.D or mean ± SME, and a two-tailed Student’s t test was used to calculate the significance of differences between the two groups. One way ANOVA was used to calculate the significance of differences among multiple groups. Kaplan–Meier survival curves were generated and compared using the log-rank test. All experiments were repeated more than twice. Statistical significance was defined as a *p <0.05, **p <0.01, ***p <0.001, **** *p* <0.0001 or nonsignificant (ns).

### Supplementary information

SUPPLEMENTAL MATERIAL

Original Data File

aj-checklist

## Data Availability

The original data of the article can be obtained from the corresponding author upon reasonable request. The RNA-seq raw data has been deposited in NCBI under accession number PRJNA1034517.
