# Supplementary material for: Carboxypeptidase A4 negatively regulates HGS-ETR1/2-induced pyroptosis by forming a positive feedback loop with the AKT signalling pathway
Source: Cell Death Dis. 2023 Dec 4;14(12):793. doi: 10.1038/s41419-023-06327-5 (PMC10696061; doi:10.1038/s41419-023-06327-5)
Supplement: Supplementary file 1 — SUPPLEMENTAL MATERIAL [file 41419_2023_6327_MOESM1_ESM.docx]

**SUPPLEMENTAL MATERIAL**


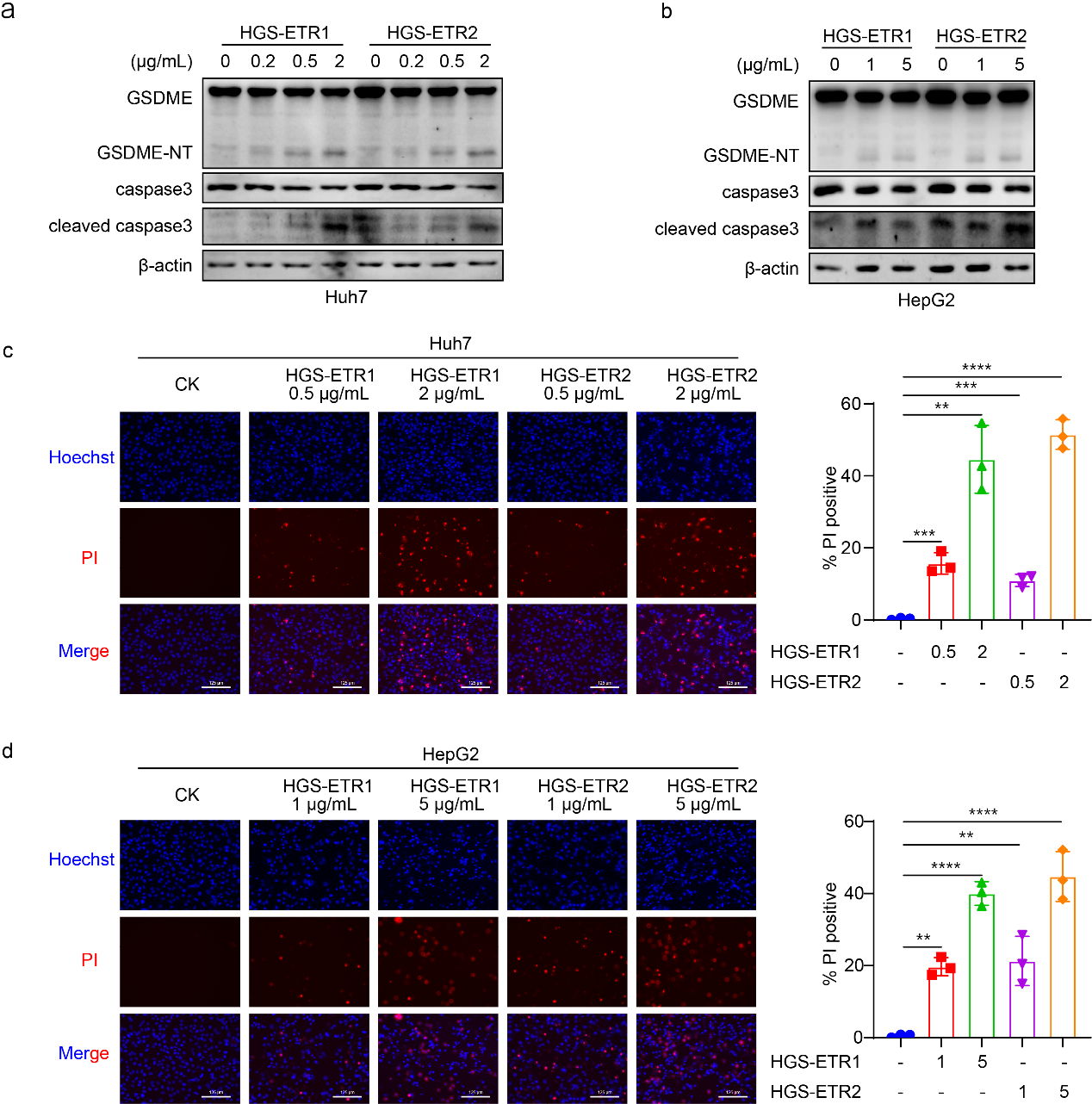


**Fig. S1 HGS-ETR1/2 induces pyroptosis mediated by cleavage of GSDME.**

**a, b** After adding different concentrations of HGS-ETR1/2 to Huh7 (**a**) or HepG2 (**b**) cells for 8 h, the suspended dead cells in the medium were collected by centrifugation and lysed together with adherent cells to obtain total proteins. The expression levels of GSDME, caspase-3 and cleaved caspase-3 were detected by western blot, and β-actin was used as an internal reference protein.

**c, d** After Huh7 (**c**) or HepG2 (**d**) cells were treated with different concentrations of HGS-ETR1/2 for 8 h, PI/Hoechst stain was added to the cell supernatant, and cell death was detected by fluorescence microscopy. The PI-positive cells (dead cells) showed red fluorescence. Scale bar, 125 μm.


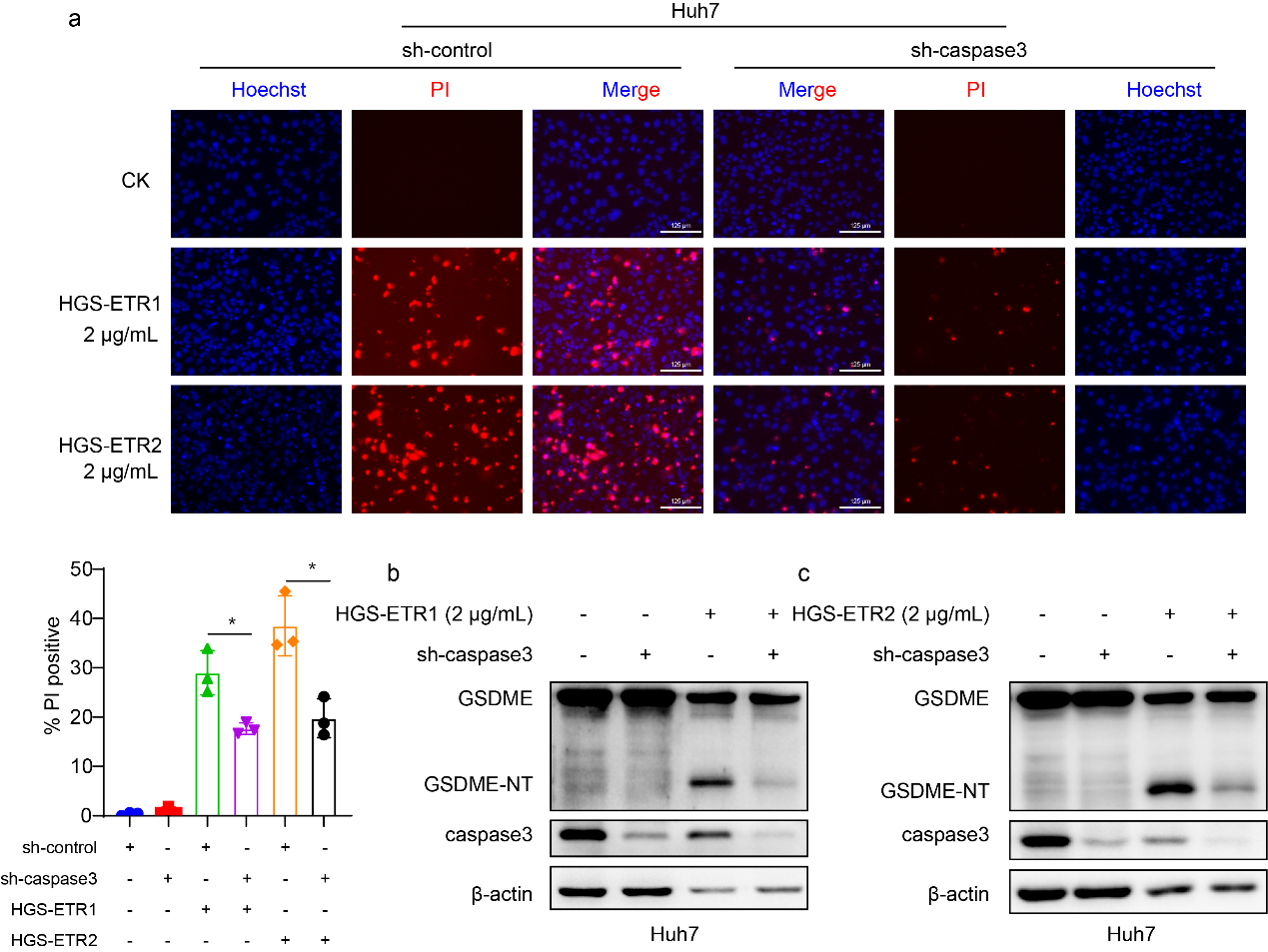


**Fig. S2 Silencing caspase-3 in Huh7 cells inhibited the pyroptosis induced by HGS-ETR1/2.**

**a** The lentivirus-silenced Huh7 cell line with caspase-3 was screened and then treated with HGS-ETR1/2 for 10 hours. PI/Hoechst staining was added to the cell supernatant, and fluorescence microscopy photos were taken to detect cell death. Scale bar, 125 μm.

**b, c** The lentivirus-silenced Huh7 cell line with caspase-3 was screened, and proteins were extracted 10 h after the addition of HGS-ETR1 (**a**) and HGS-ETR2 (**b**). The expression levels of GSDME and caspase-3 were detected by western blot, and β-actin was used as the internal reference protein.


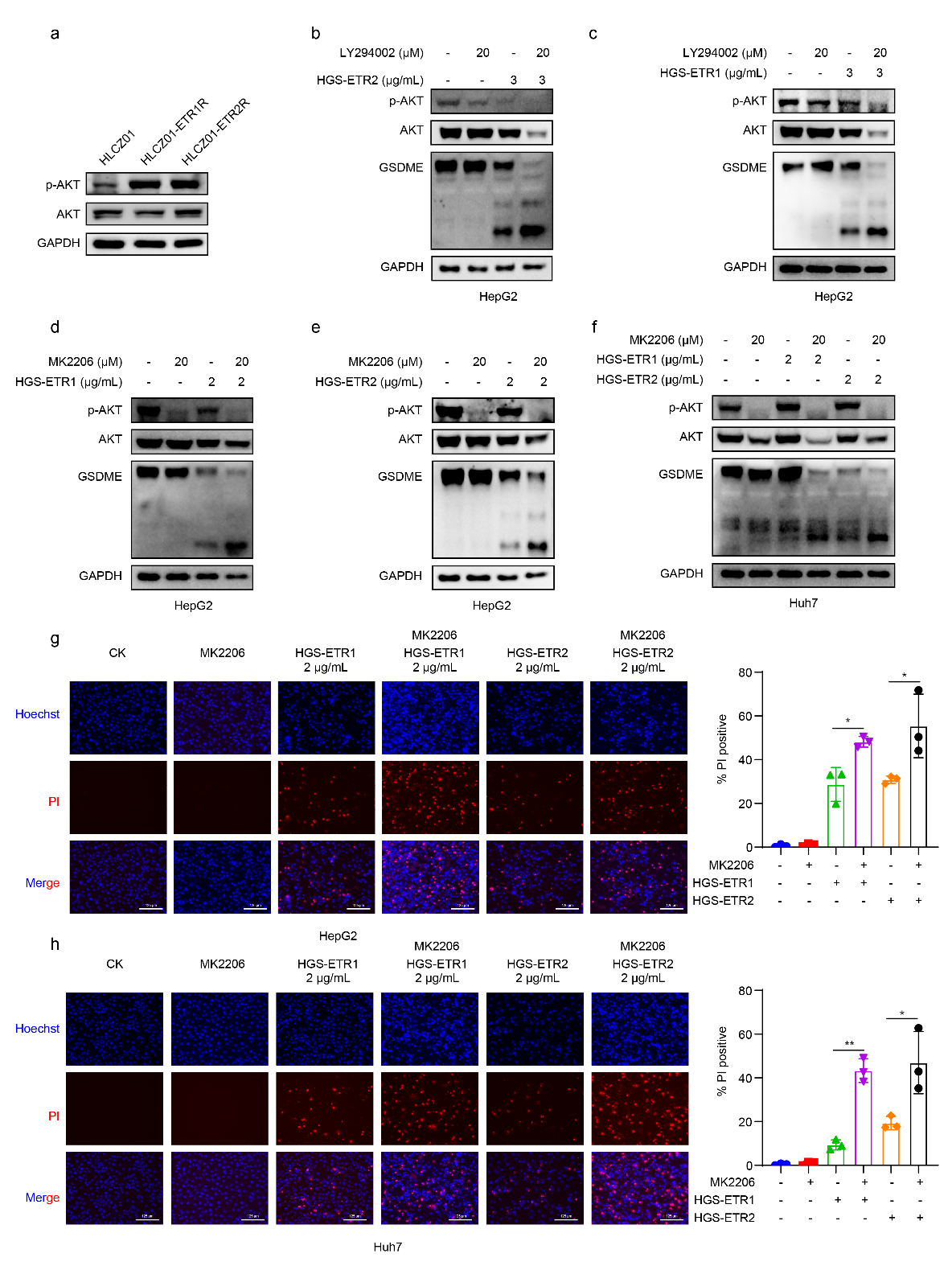


**Fig. S3 The AKT signalling pathway regulates pyroptosis induced by HGS-ETR1/2.**

**a** western blot confirmed the upregulated expression of p-AKT in HGS-ETR1/2R cells, using GAPDH as the internal reference protein.

**b, c** LY294002 was added to HepG2 cells, and after 1 h, HGS-ETR1 (**b**) or HGS-ETR2 (**c**) was added to HepG2 cells for 8 h. Proteins were collected, and GSDME cleavage was detected.

**d-f** HepG2 (**d, e**) and Huh7 (**f**) cells were treated with MK2206 for 1 h, followed by HGS-ETR1/2 for 8 h, and proteins were collected to detect the cleavage of GSDME.

**g, h** HepG2 (**g**) and Huh7 (**h**) cells were treated with MK2206 for 1 h, followed by HGS-ETR1/2 for 8 h. PI and Hoechst dye were added to the cell culture medium and incubated for 20 min. Cell death was recorded by fluorescence photography. Scale bar, 125 μm.


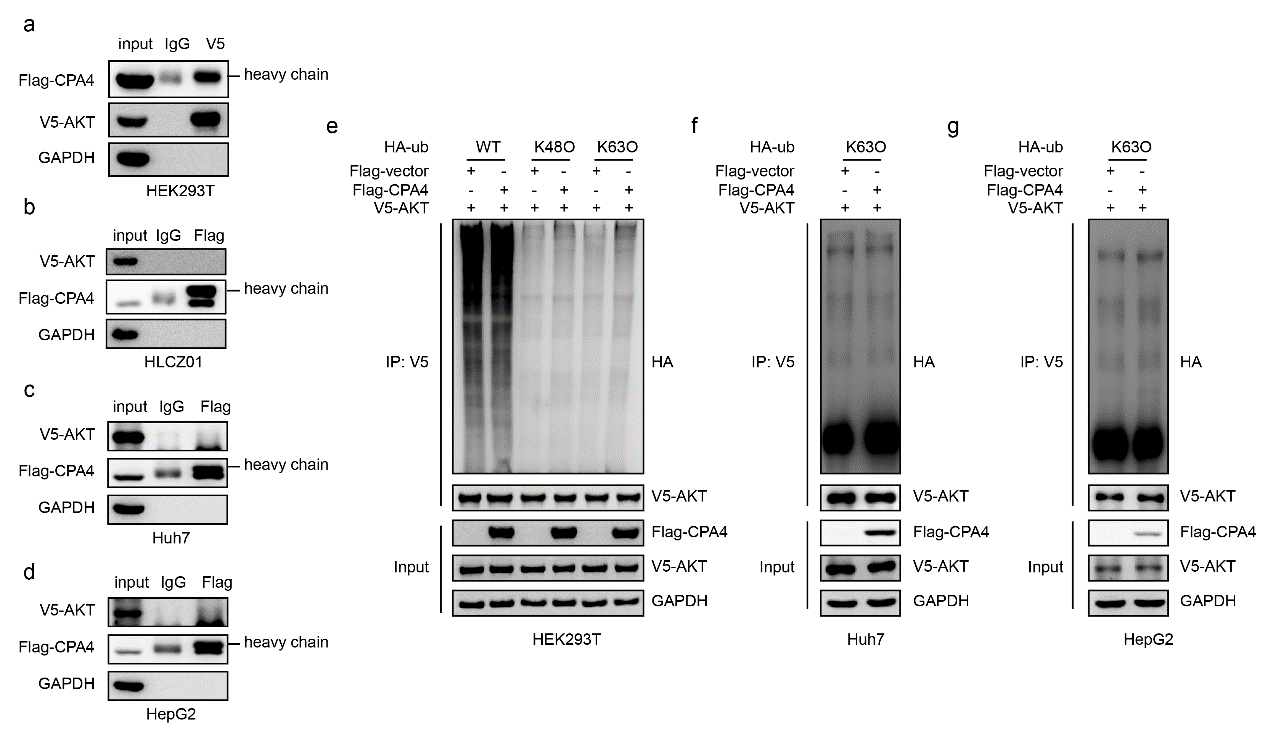


**Fig. S4 There is no direct interaction between CPA4 and AKT, and CPA4 does not affect K63 ubiquitination of AKT.**

**a-d** V5-AKT and flag-CPA4 were overexpressed in HEK293T (**a**), HLCZ01 (**b**), Huh7 (**c**) and HepG2 (d) cells, and no interaction was detected when V5-AKT or flag-CPA4 proteins were pulled down by anti-flag (**a**) or anti-V5 antibodies (**b-d**).

**e-g** The regulation of AKT K63 ubiquitination by CPA4 was detected in HEK293T (**e**), Huh7 (**f**) and HepG2 (**g**) cells, and it was confirmed that CPA4 did not affect AKT K63 ubiquitination.


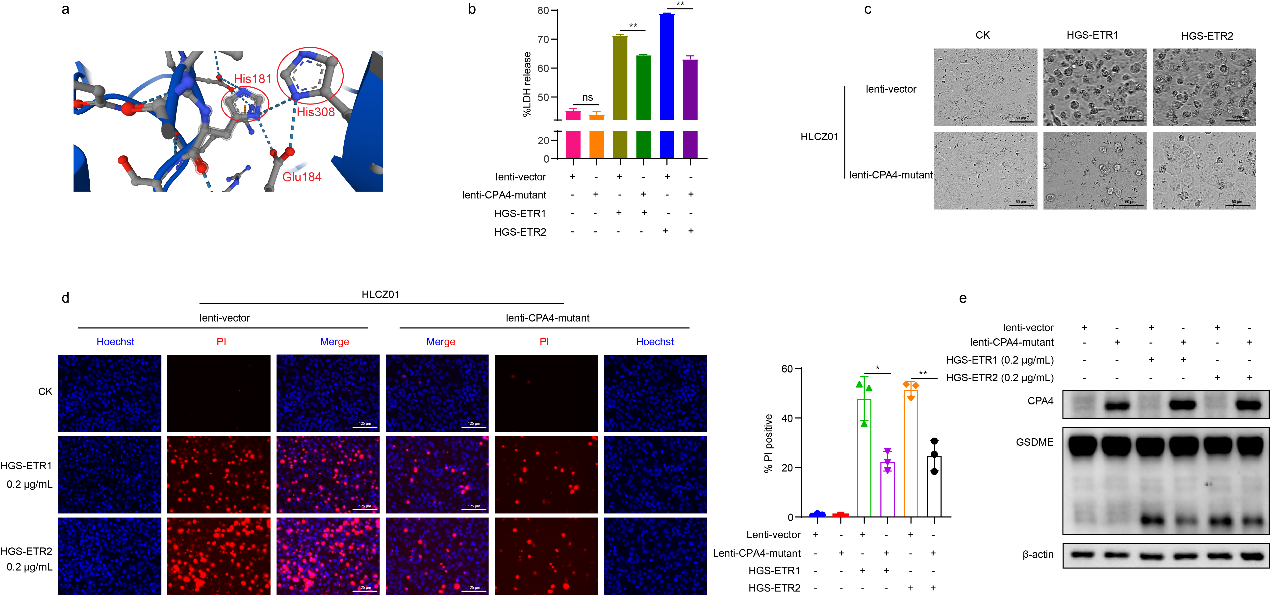


**Fig. S5 CPA4 can regulate pyroptosis independently of its carboxypeptidase activity.**

**a** Structure diagram of the CPA4 zinc ion binding site (<https://alphafold.ebi.ac.uk/> ). His181, Glu184, and His308 were zinc ion binding sites of CPA4, and CPA4-mutant mutant all His181 and His308 into Ala.

**b** Stable cell lines infected with HLCZ01 expressing the CPA4-mutant were screened and treated with 0.2 μg/mL of GHS-ETR1/2 for 8 h. The cell supernatant was collected to detect the release of LDH.

**c** After adding 0.2 μg/mL of HGS-ETR1/2 to HLCZ01 stable cell lines of lentivirus-infected CPA4-mutant for 8 h, the cell pyroptosis morphology was observed with a microscope.

**d** 0.2 μg/mL of HGS-ETR1/2 was added to the HLCZ01 stable CPA4-mutant cell lines infected with lentivirus. PI and Hoechst dye were added 8 h later, and the cell death was observed by fluorescence photography. Scale bar, 125 μm.

**e** 0.2 μg/mL of HGS-ETR1/2 was added to stable cell lines of lentivirus-infected CPA4-mutant. After 8 h, proteins were collected and GSDME cleavage was detected, with β-actin as the internal reference.

The experiment was independently replicated three times. A two-sided Student’s t test was used to analyse significant differences. (*** p＜0.001).


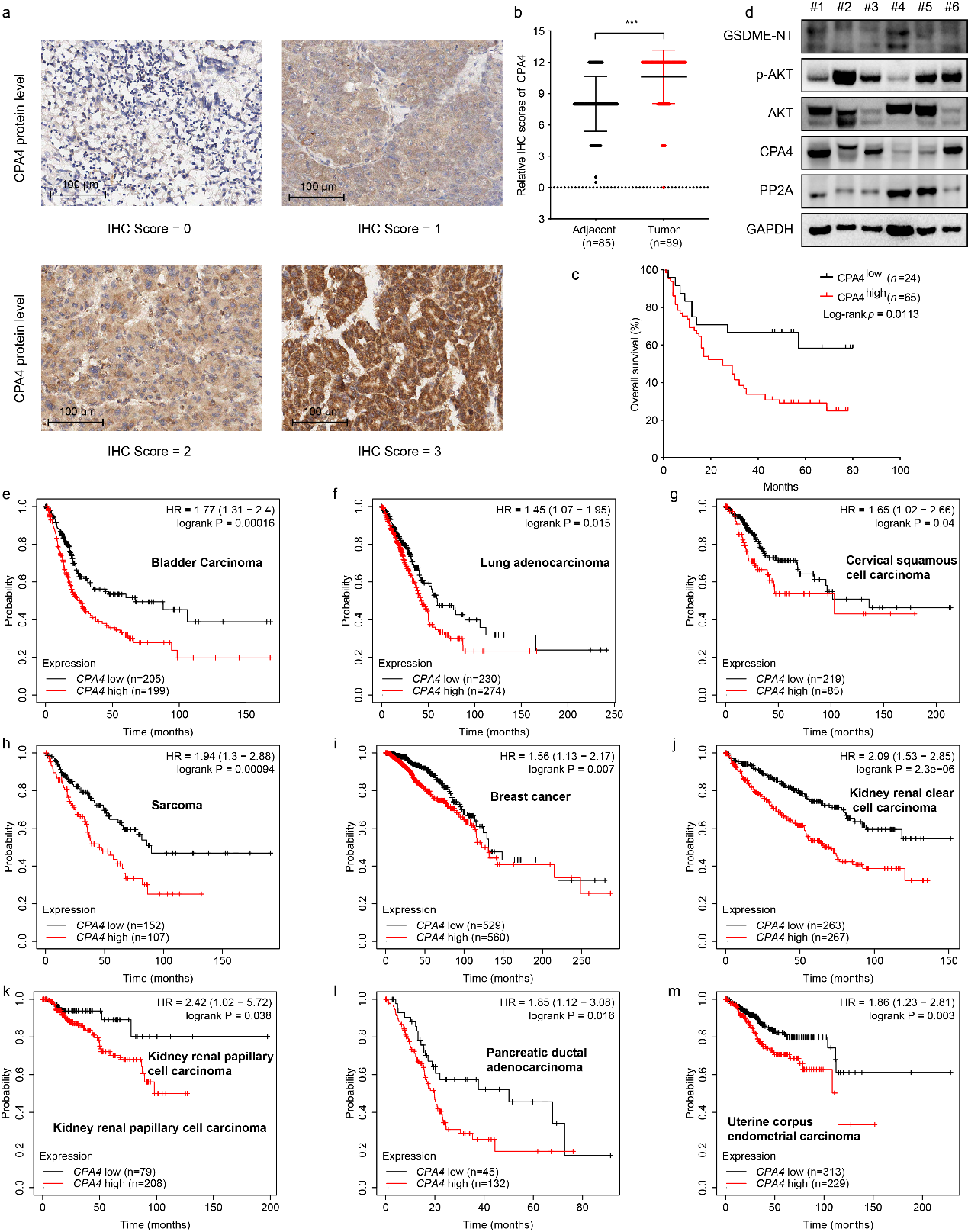


**Fig. S6 CPA4 is associated with poor tumour prognosis in the clinic.**

**a** CPA4 antibody was used to perform immunohistochemistry on the tissue chip of the patient. The evaluation criteria for the colouring depth were 0 for negative, 1 for low expression, 2 for moderate expression, and 3 for high expression. Scale bar, 100 μm.

**b** The expression of CAP4 protein in tumour tissues (n= 89) and adjacent tissues (n=85) showed that the expression of CAP4 protein in tumor tissues was significantly higher than that in adjacent tissues. A two-sided Student’s t test was used to analyse significant differences. (*** p＜0.001).

**c** Kaplan-Meier analysis was used to analyse the relationship between CPA4 and the survival rate of patients with liver cancer. High CPA4 expression is associated with poor prognosis in patients with liver cancer.

**d** The liver cancer tissue protein of the patient (n=6) was extracted, and western blot was used to detect the expression of CAP4, PP2A, AKT, p-AKT and GSDME, with GAPDH as the internal reference protein.

**e-j** Kaplan-meier plotter (<http://kmplot.com/analysis/index.php?p=background>) was used to analyse the relationship between the expression level of CPA4 and the survival rate of patients with various cancers.

**Table S1 Primers used in vector construction.**

| **Vector** | **Forward Primer (****5’→3’)** | **Reverse Primer (5’→3’)** |
| --- | --- | --- |
| Lenti-*CPA4* | GCTCTAGAGCATGAGGTGGATACTGTTCATTGGG | AAGGAAAAAAGCGGCCGCAAAAGGAAAACTAGTAGAGGTTGTCCCGCA |
| Flag-*CPA4* | AAGGAAAAAAGCGGCCGCAAAAGGAAAAATGAGGTGGATACTGTTCAT | GCTCTAGAGCGGTAGAGGTTGTCCCGCACATGCTCC |

**Table S2 Primers used in qPCR assays.**

| **Name** | **Forward Primer (5’→3’)** | **Reverse Primer (5’→3’)** |
| --- | --- | --- |
| *GSDME* | ACATGCAGGTCGAGGAGAAGT | TCAATGACACCGTAGGCAATG |
| *CPA4* | AGGTGGATACTGTTCATTGGGG | TTGCTGATCTCGTCTCCATTTC |
| *DR4* | ACCTTCAAGTTTGTCGTCGTC | CCAAAGGGCTATGTTCCCATT |
| *DR5* | GCCCCACAACAAAAGAGGTC | AGGTCATTCCAGTGAGTGCTA |
| *GAPDH* | GGAGCGAGATCCCTCCAAAAT | GGCTGTTGTCATACTTCTCATGG |
