# Supplementary material for: Carboxypeptidase A4 negatively regulates HGS-ETR1/2-induced pyroptosis by forming a positive feedback loop with the AKT signalling pathway
Source: Cell Death Dis. 2023 Dec 4;14(12):793. doi: 10.1038/s41419-023-06327-5 (PMC10696061; doi:10.1038/s41419-023-06327-5)
Supplement: Supplementary file 2 — Original Data File [file 41419_2023_6327_MOESM2_ESM.pdf]

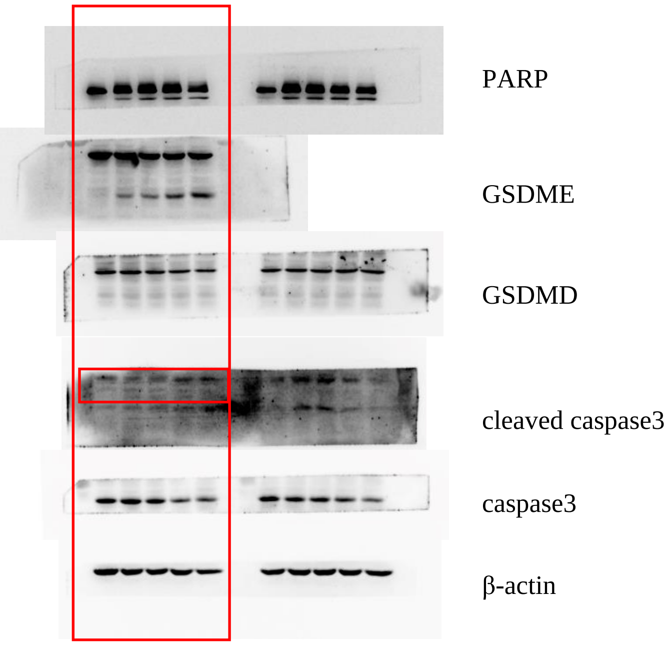

figure 1f

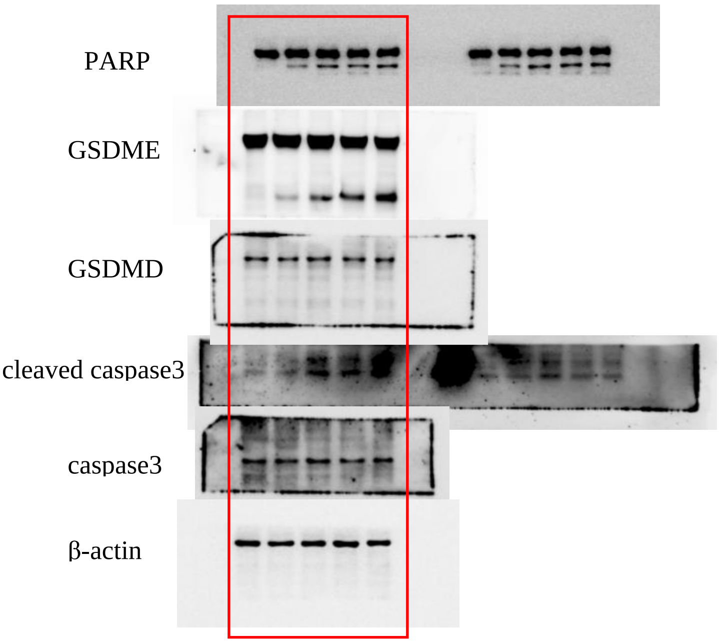

figure 1g

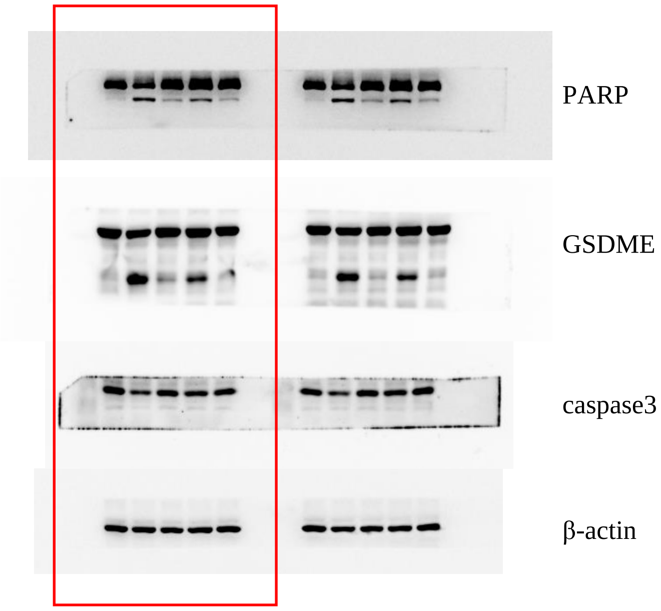

figure2d

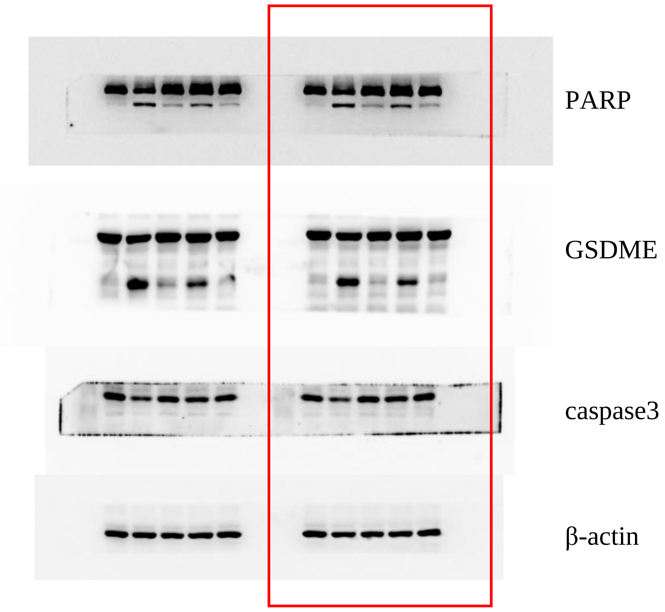

figure2h

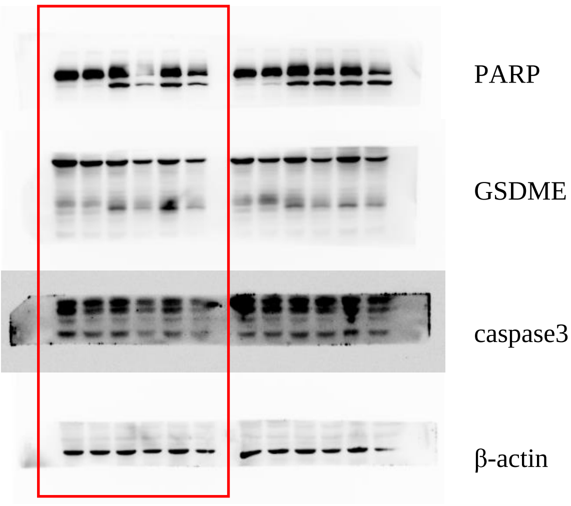

figure2i

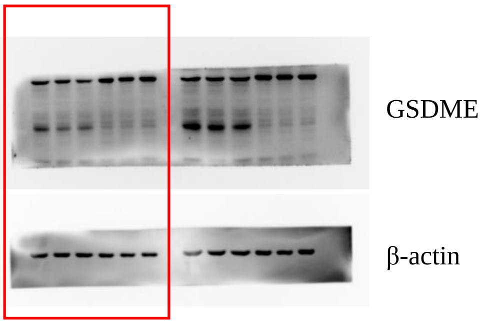

figure3b

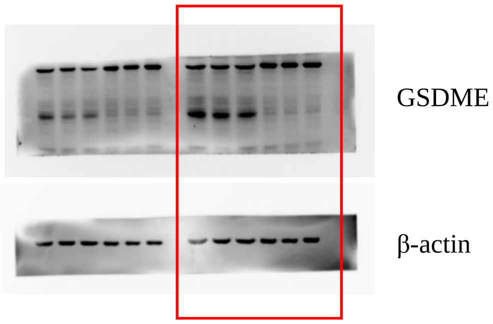

figure3c

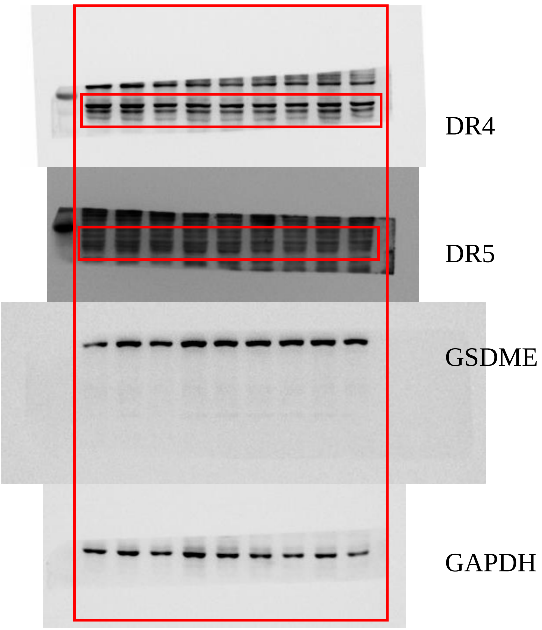

figure3e

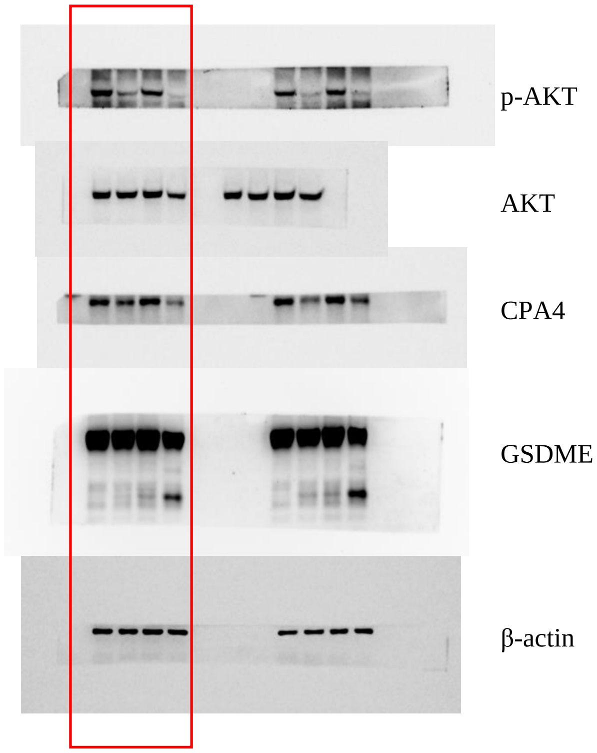

figure5a

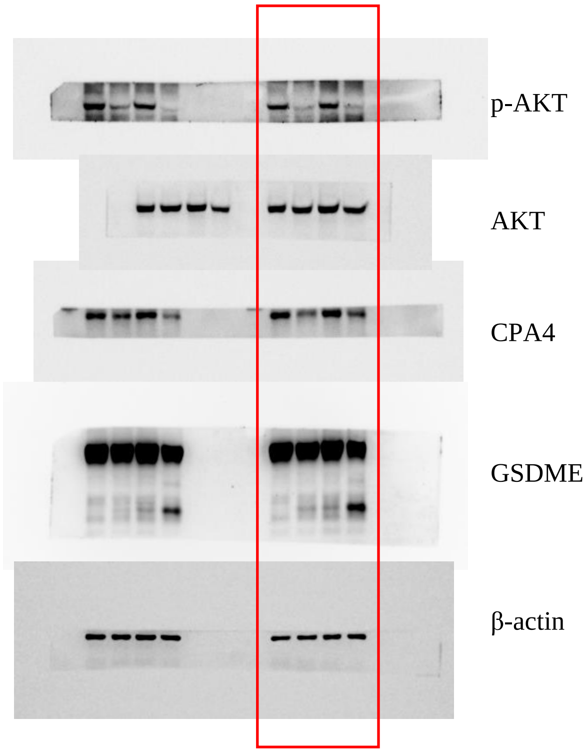

figure5b

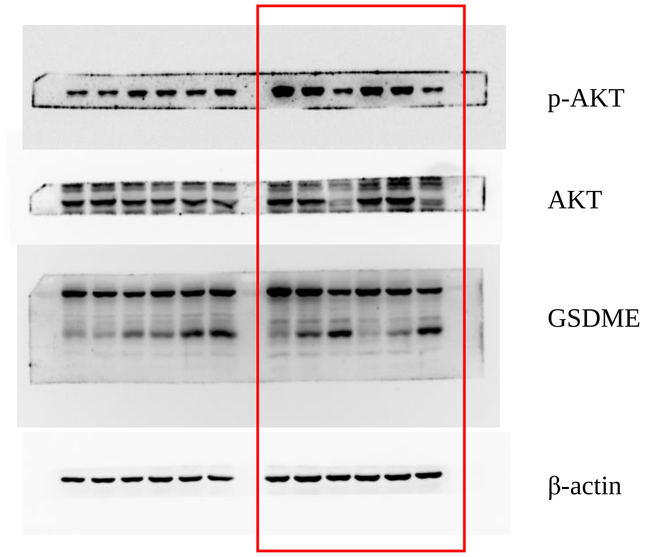

figure5c

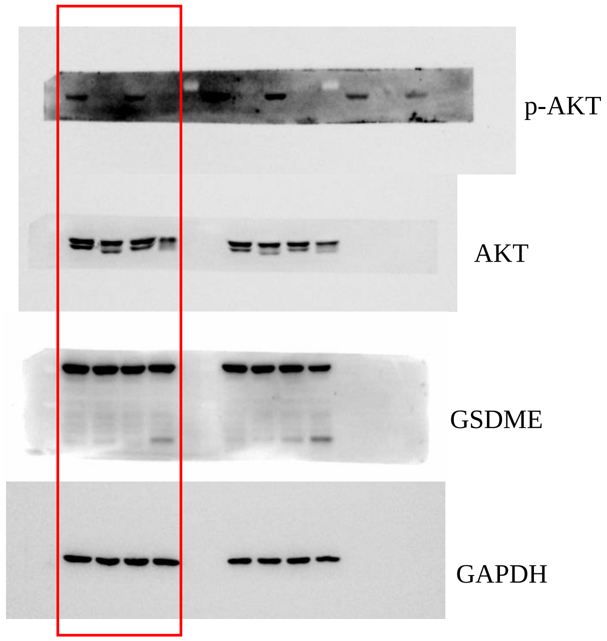

fugure5e

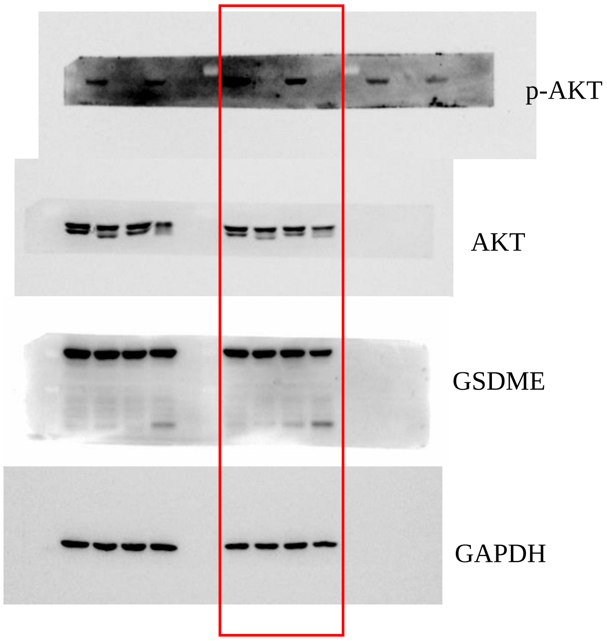

fugure5f

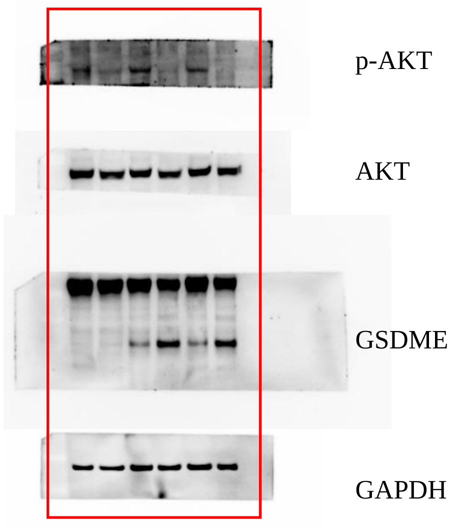

Figure 5g

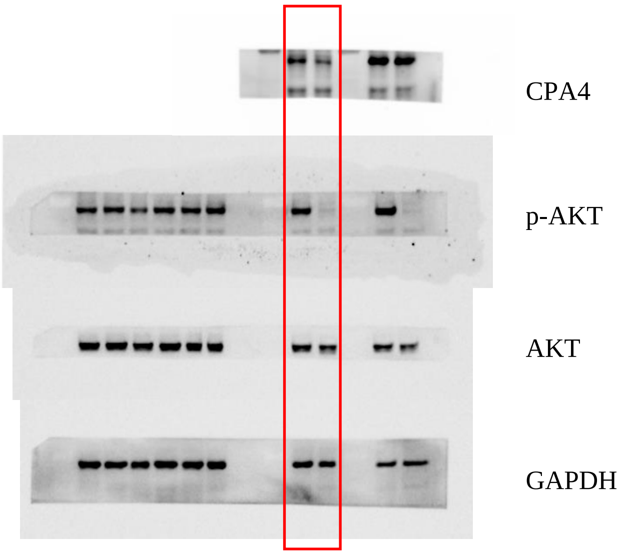

figure5m

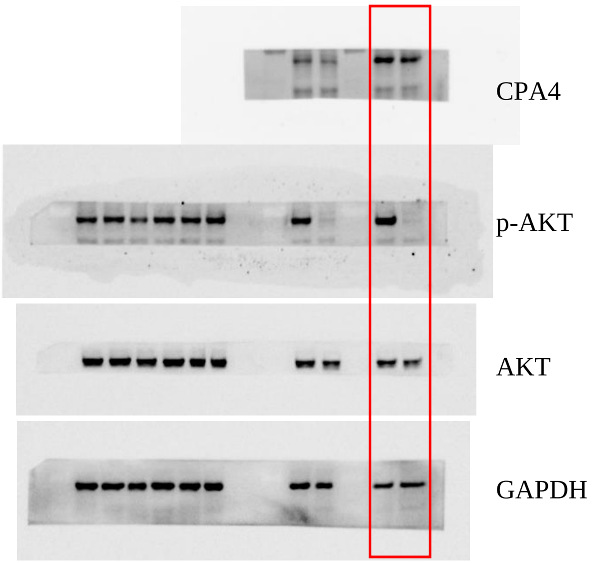

figure5n

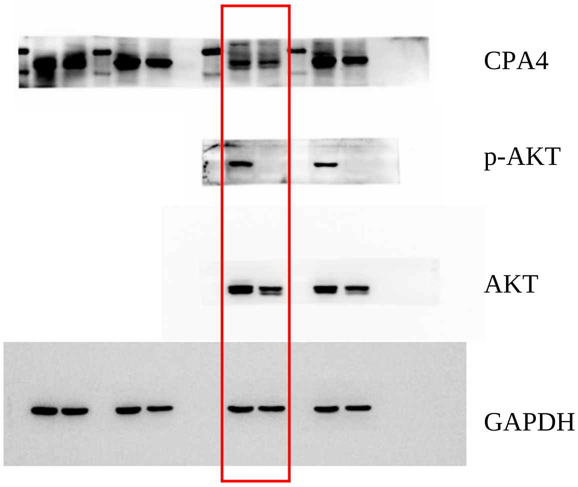

figure5q

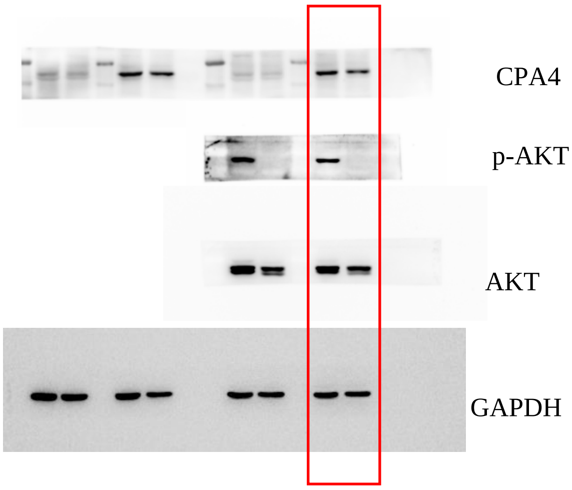

figure5r

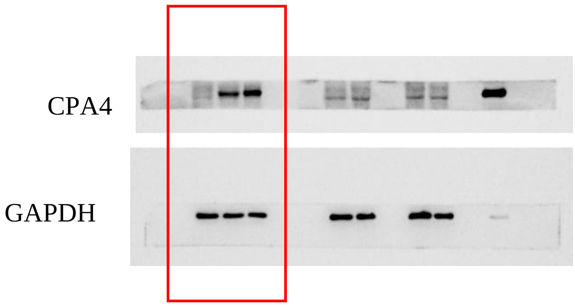

figure6b

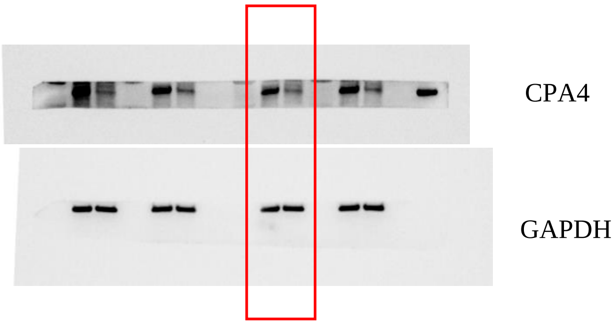

figure6d

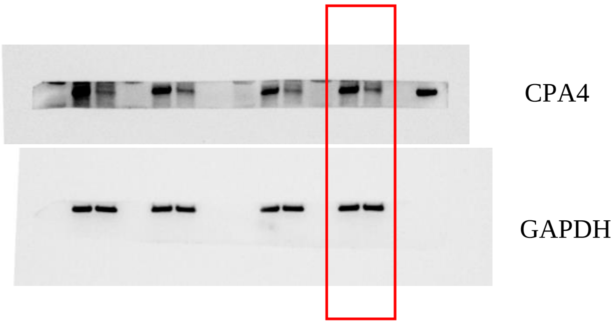

figure6f

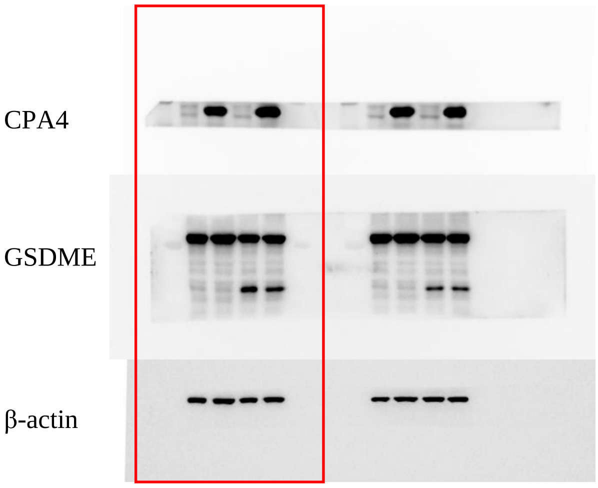

figure6j

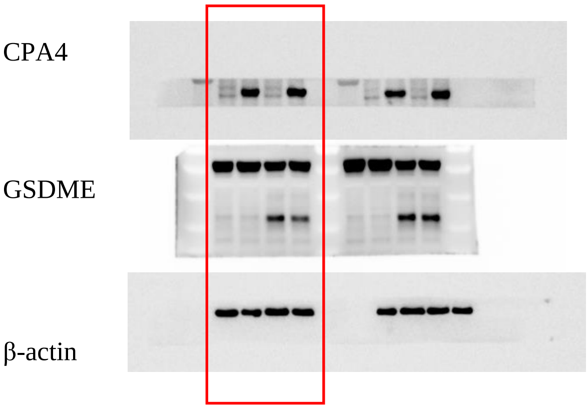

figure6k

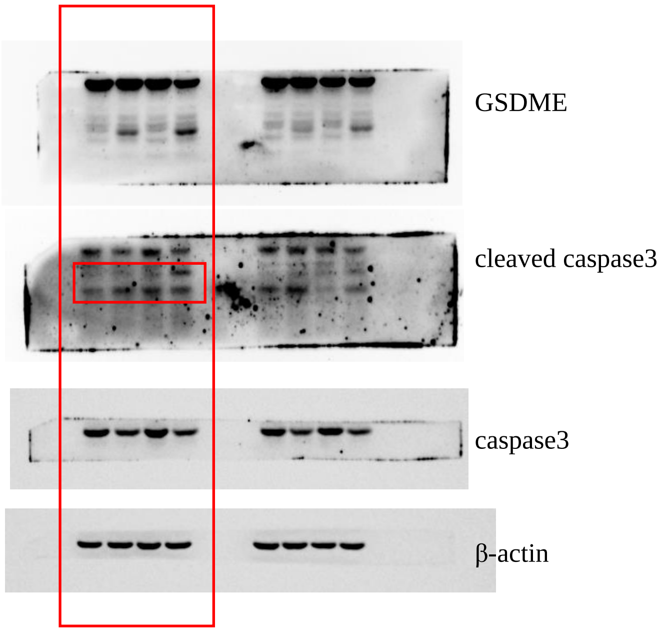

figure6g

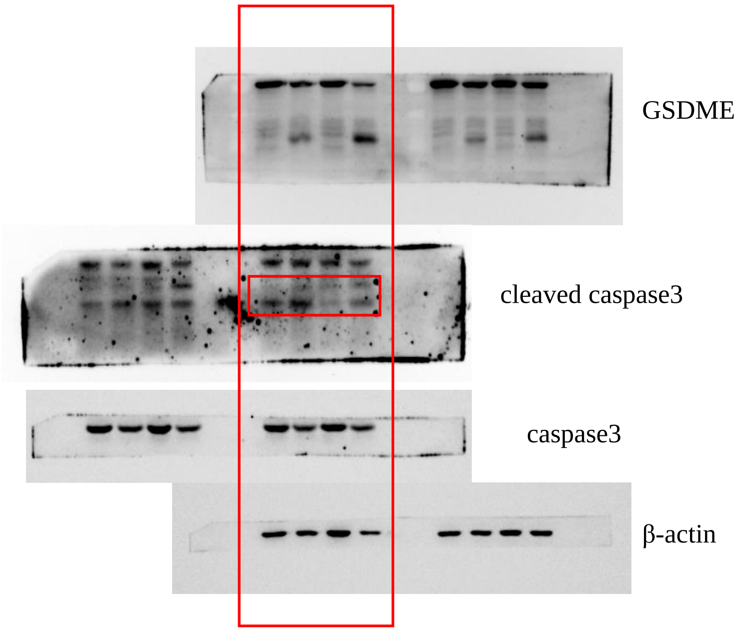

figure6h

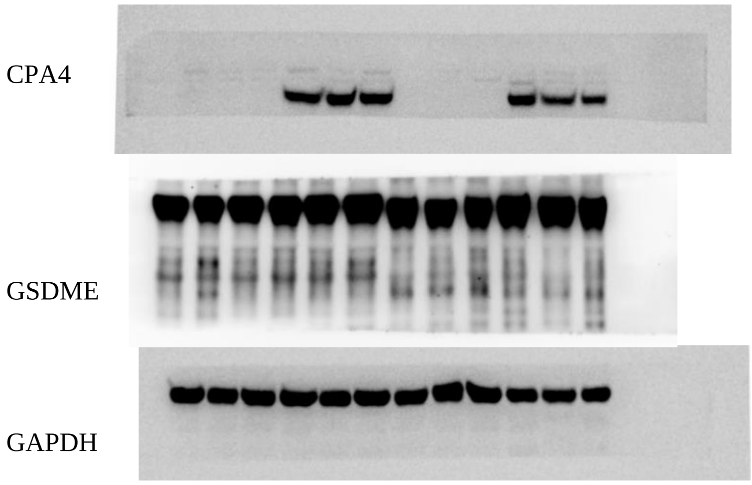

figure 6s

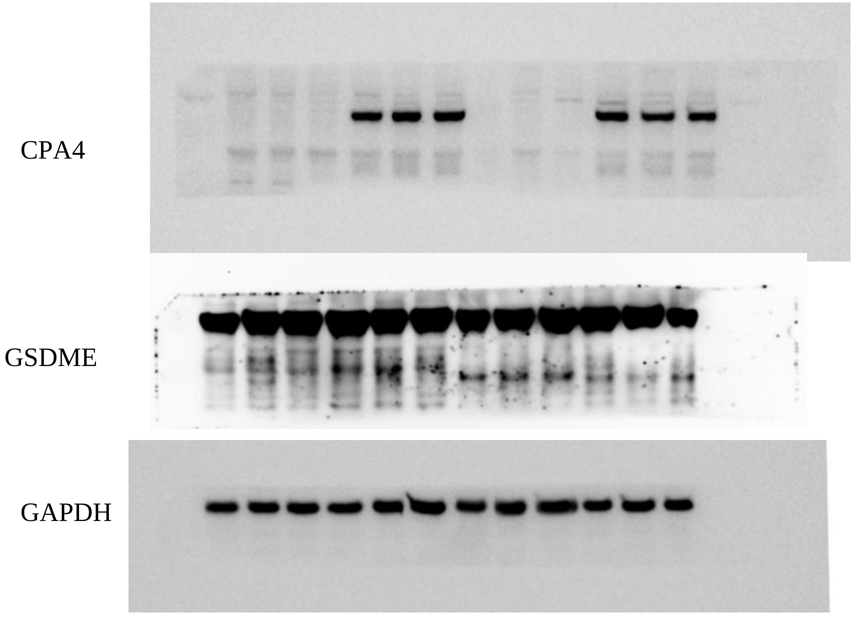

figure 6t

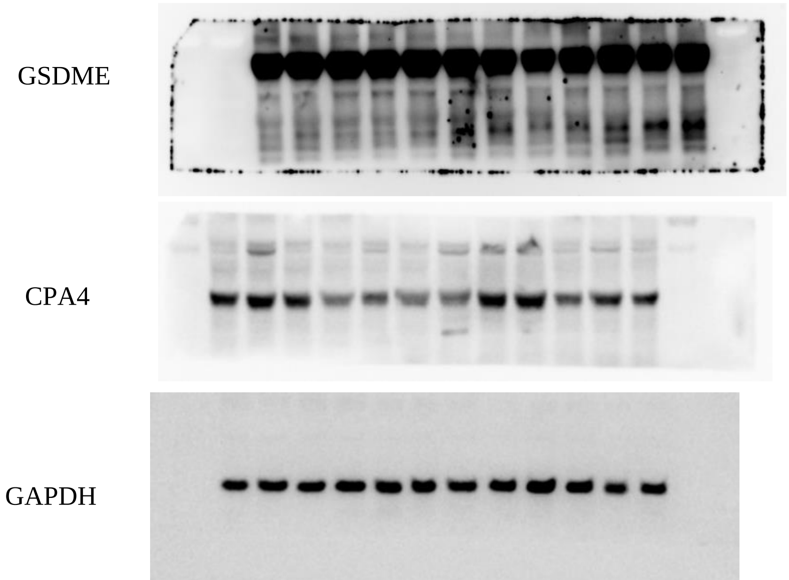

figure 6y

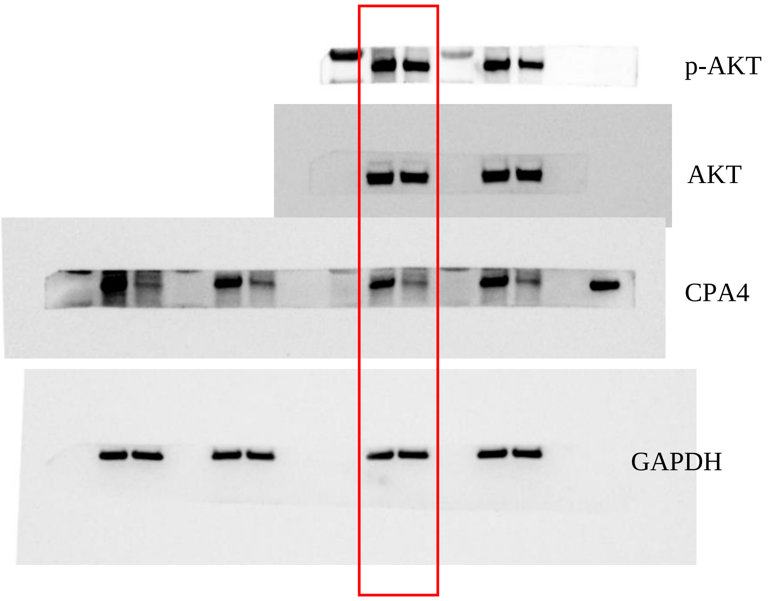

figure7a

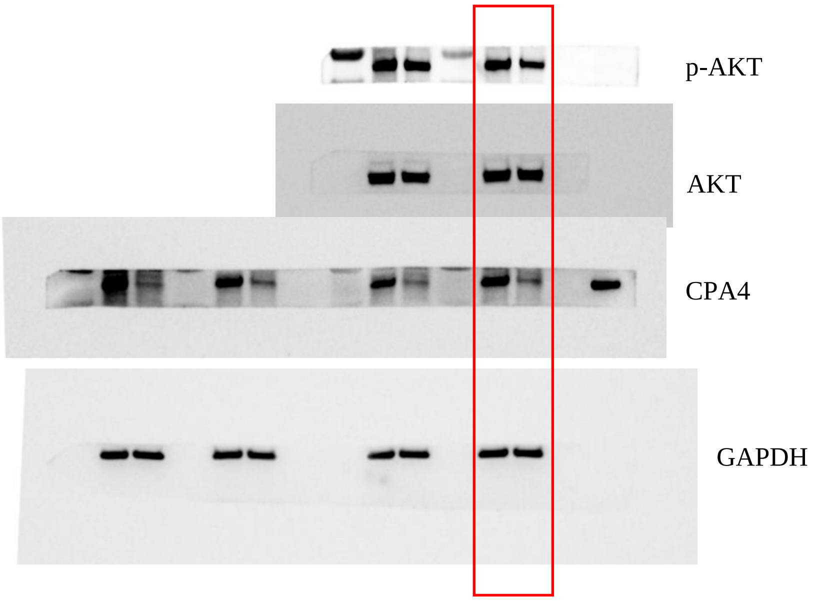

figure7b

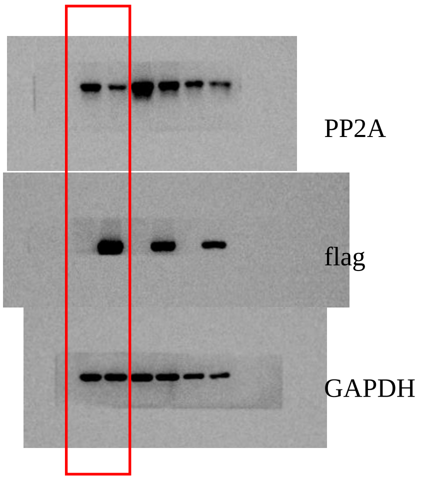

figure7c

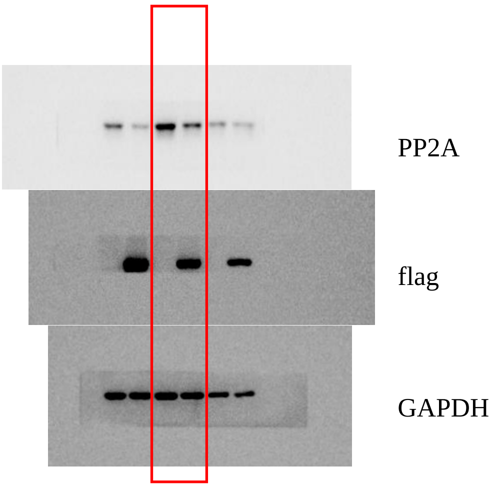

figure7d

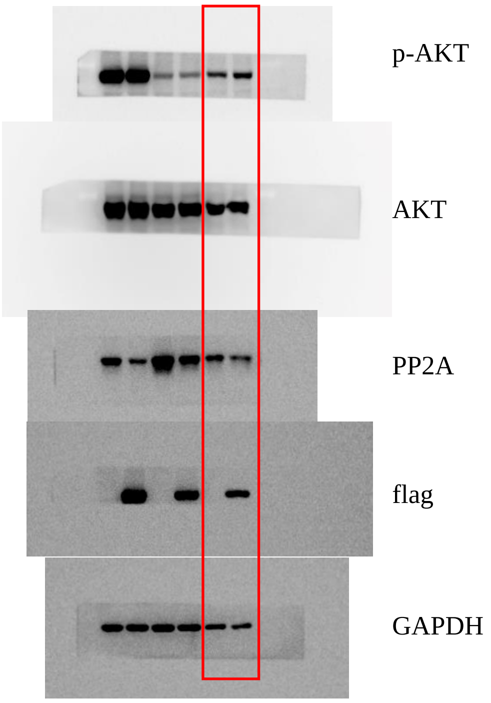

figure7e

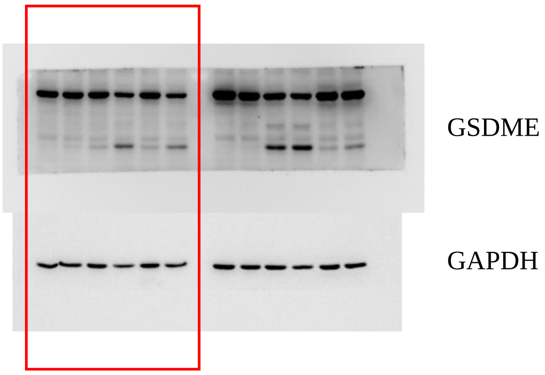

figure7f

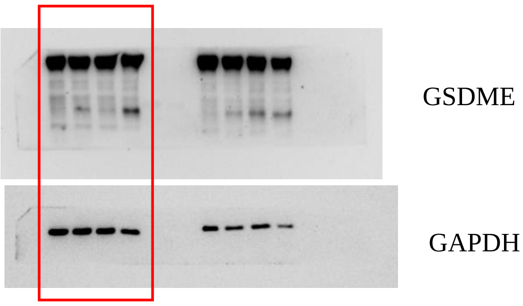

figure7g

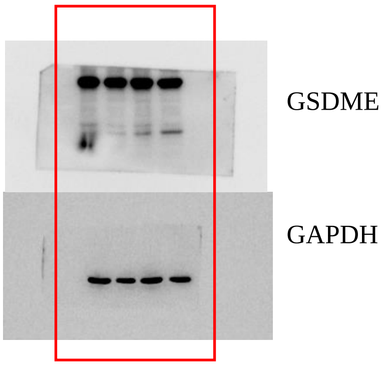

figure7h

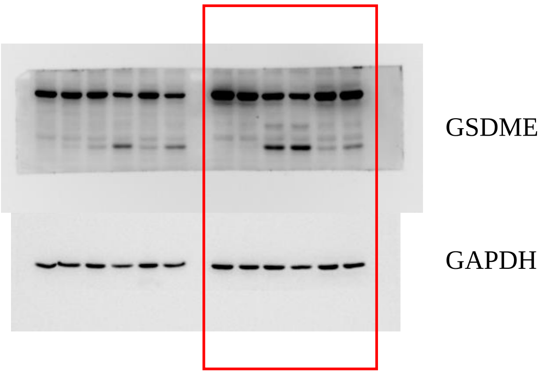

figure7k

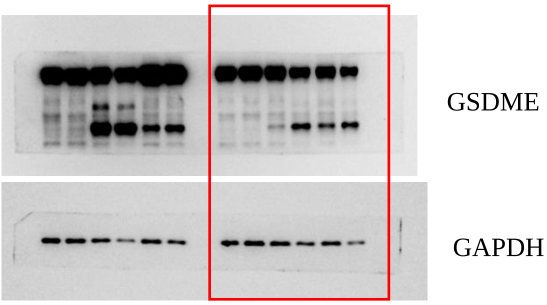

figure7l

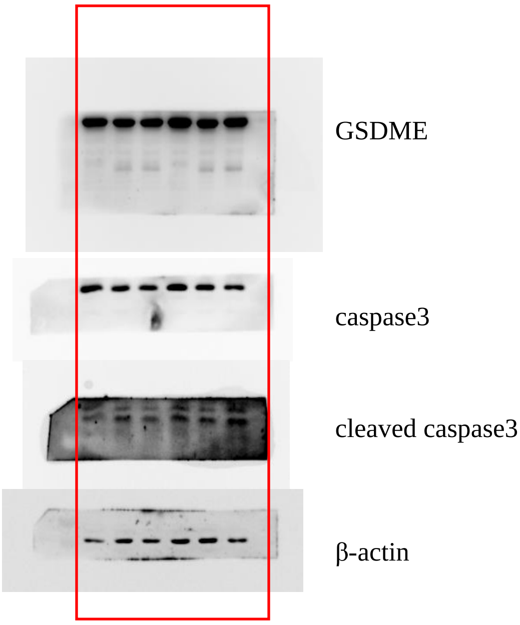

figureS1b

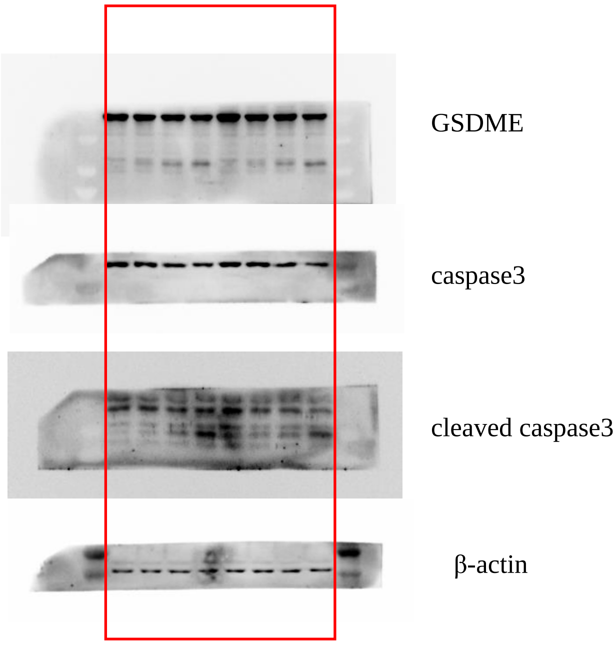

figureS1a

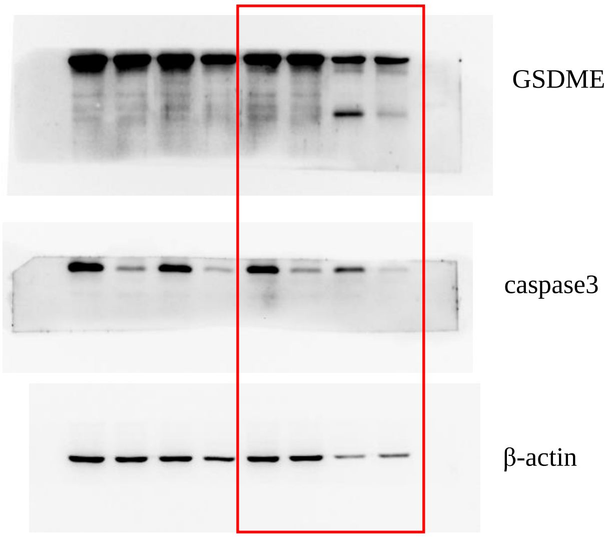

figureS2b

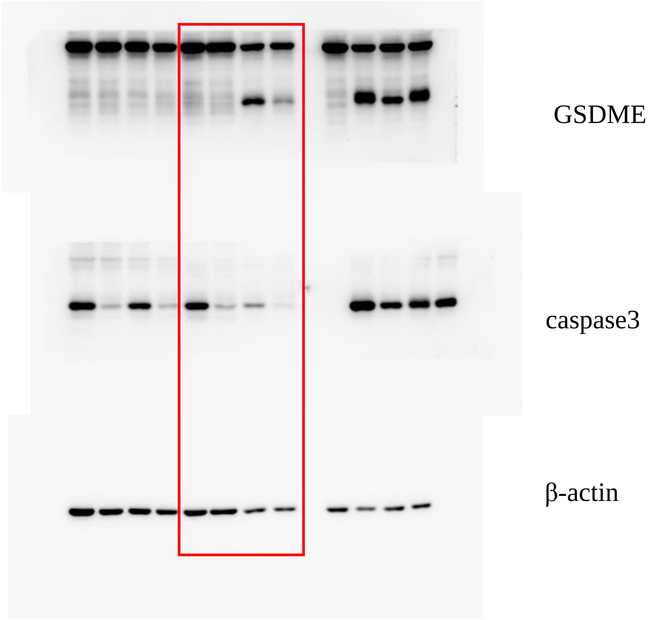

figureS2c

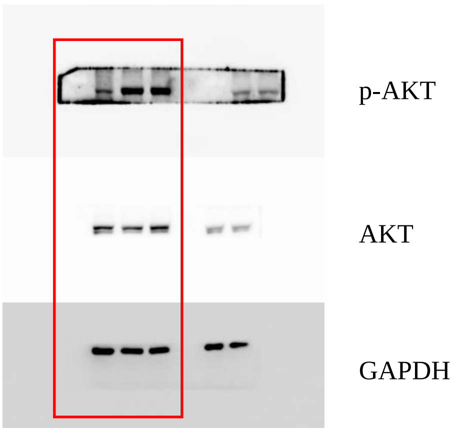

figureS3a

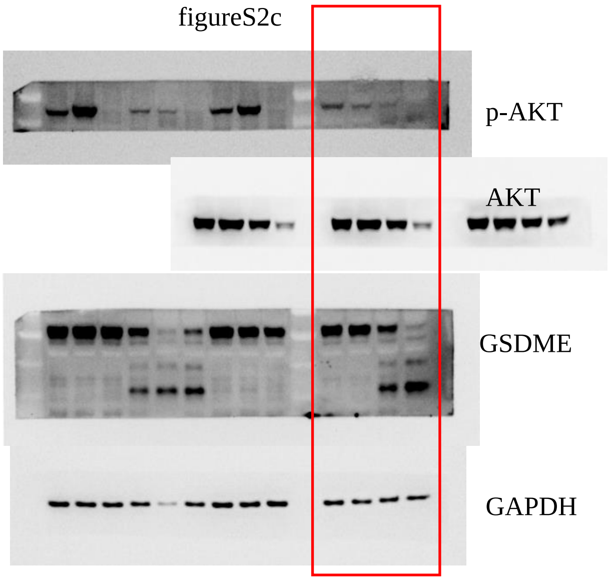

figureS3b

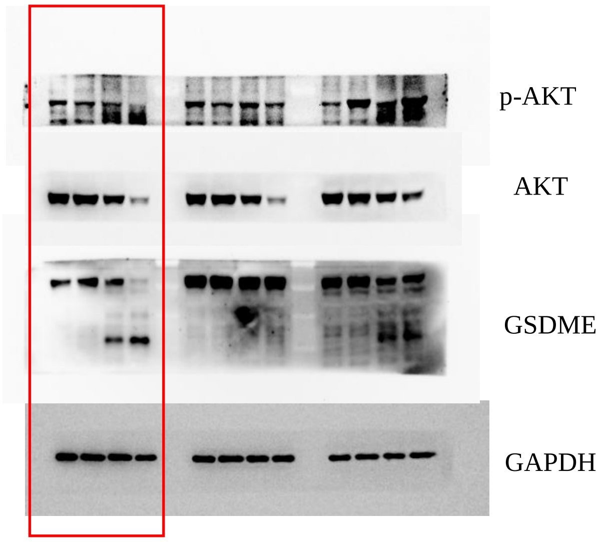

figureS3c

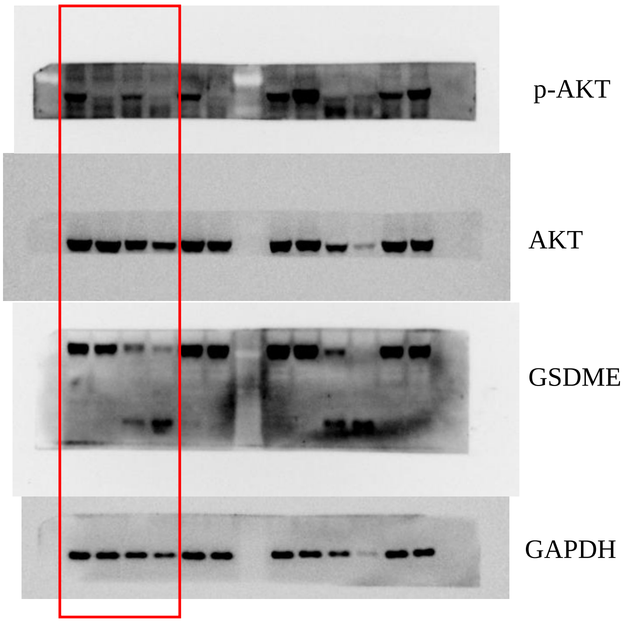

figureS3d

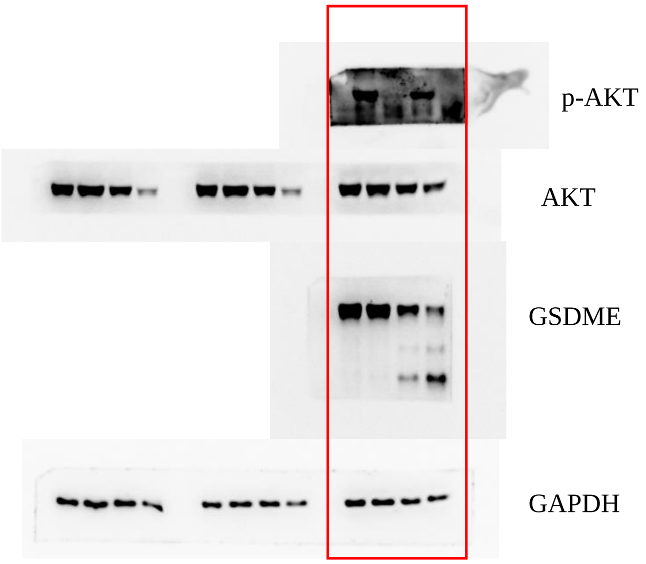

figureS3e

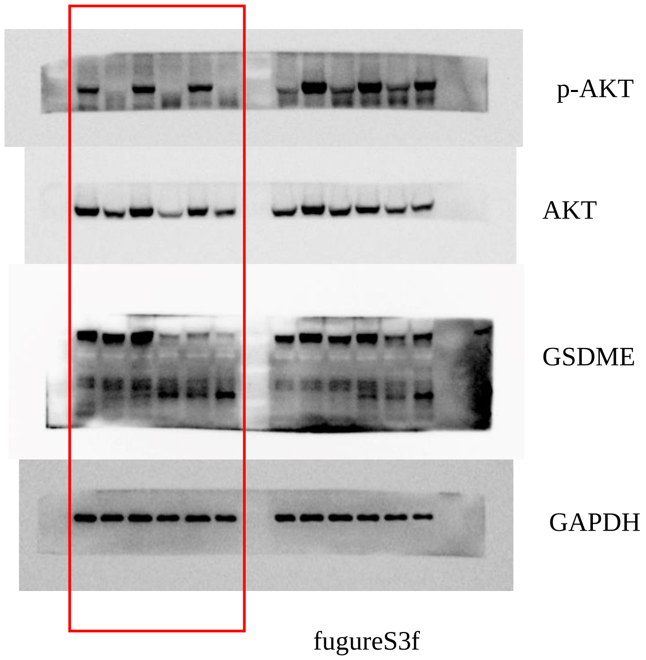

fugureS3f

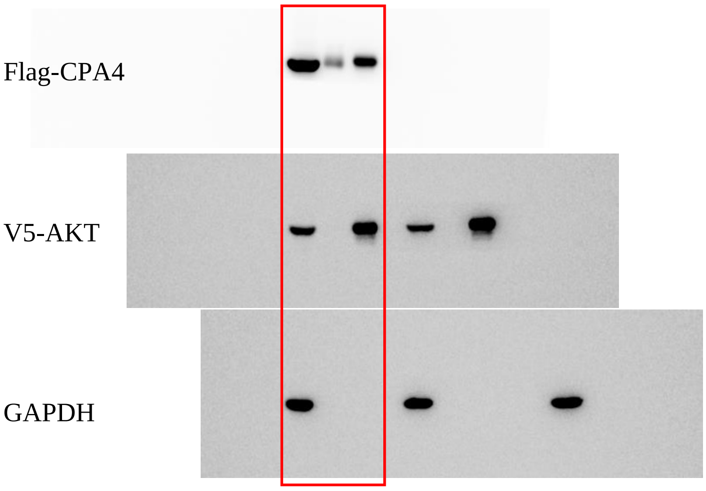

figureS4a

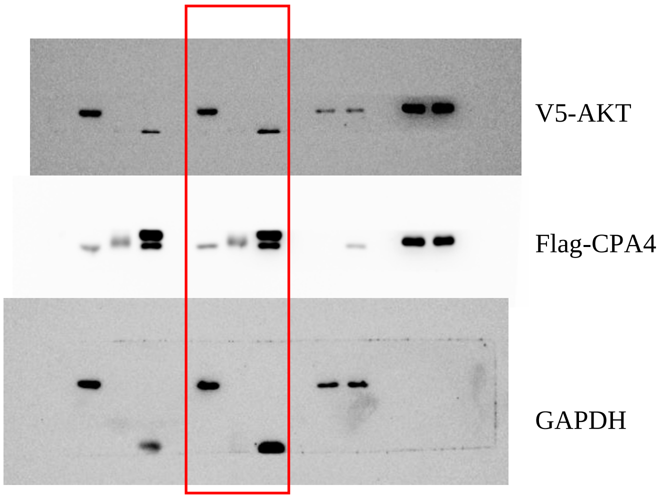

figureS4b

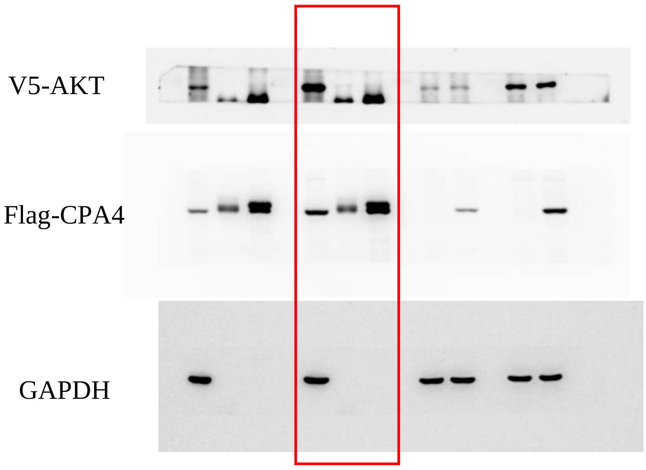

figureS4c

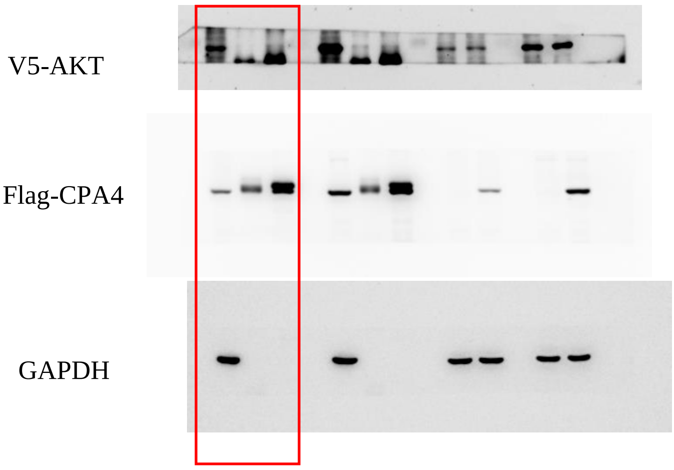

figureS4d

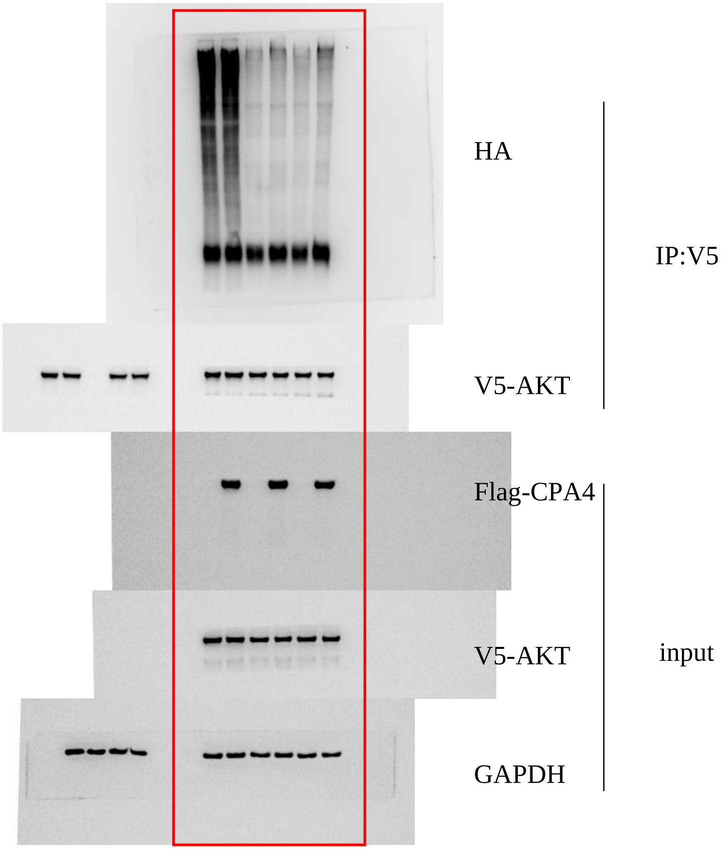

figureS4e

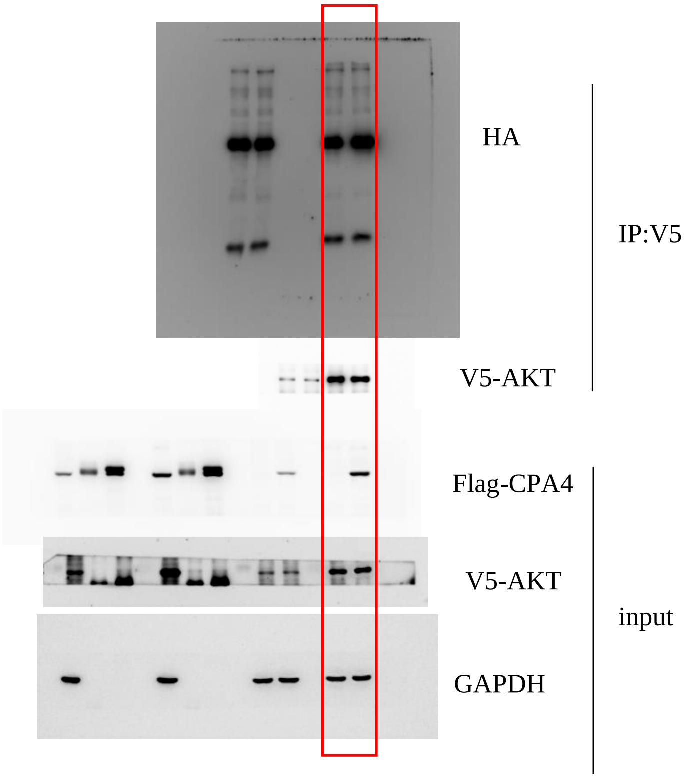

figureS4f

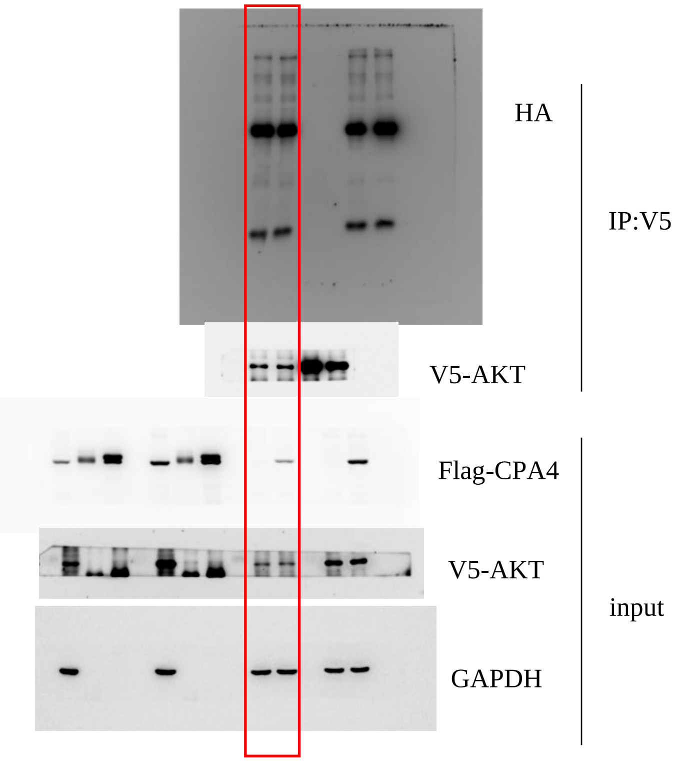

figureS4g

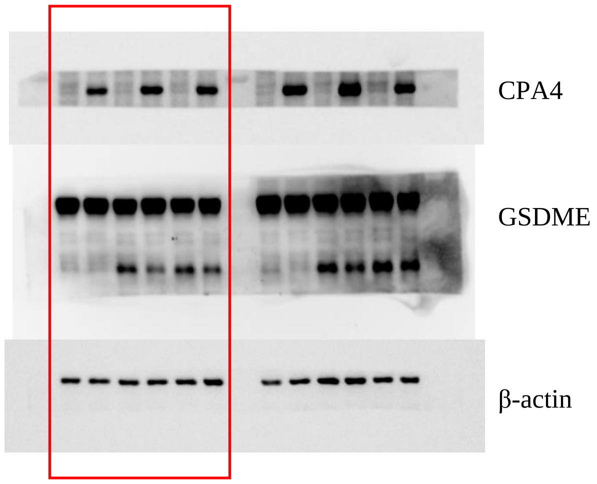

figureS5e

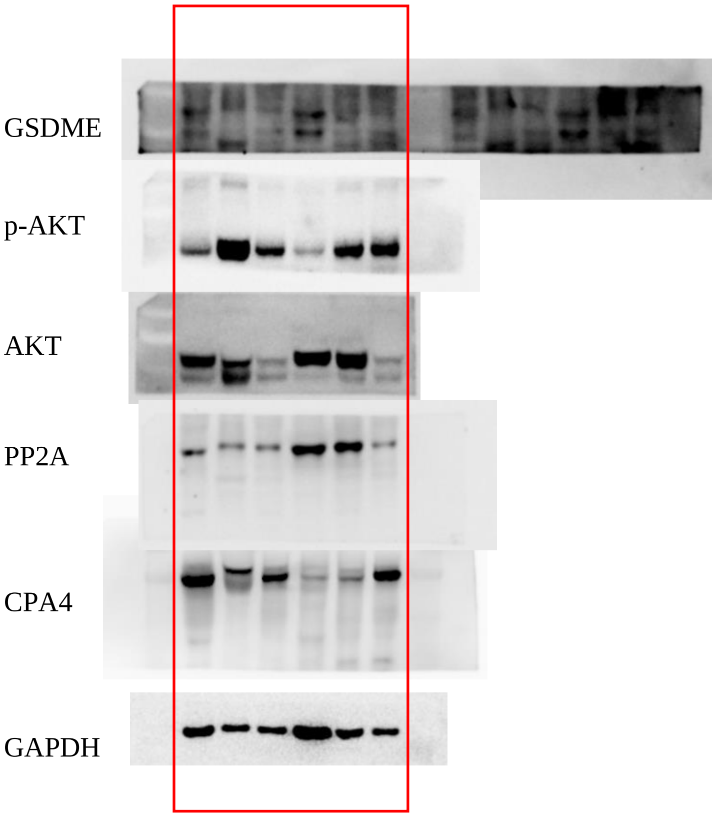

figureS6d
